# Evolution of the tRNA^Leu^ (UAA) Intron and Congruence of Genetic Markers in Lichen-Symbiotic *Nostoc*

**DOI:** 10.1371/journal.pone.0131223

**Published:** 2015-06-22

**Authors:** Ulla Kaasalainen, Sanna Olsson, Jouko Rikkinen

**Affiliations:** 1 Department of Geobiology, University of Göttingen, Göttingen, Germany; 2 Department of Biosciences, University of Helsinki, Helsinki, Finland; Belgian Nuclear Research Centre SCK•CEN, BELGIUM

## Abstract

The group I intron interrupting the tRNALeu UAA gene (*trnL*) is present in most cyanobacterial genomes as well as in the plastids of many eukaryotic algae and all green plants. In lichen symbiotic *Nostoc*, the P6b stem-loop of *trnL* intron always involves one of two different repeat motifs, either Class I or Class II, both with unresolved evolutionary histories. Here we attempt to resolve the complex evolution of the two different *trnL* P6b region types. Our analysis indicates that the Class II repeat motif most likely appeared first and that independent and unidirectional shifts to the Class I motif have since taken place repeatedly. In addition, we compare our results with those obtained with other genetic markers and find strong evidence of recombination in the 16S rRNA gene, a marker widely used in phylogenetic studies on Bacteria. The congruence of the different genetic markers is successfully evaluated with the recently published software Saguaro, which has not previously been utilized in comparable studies.

## Introduction

Cyanobacteria form symbioses with many different types of organisms, and in terrestrial ecosystems *Nostoc* is the most common genus of symbiotic cyanobacteria. Symbiotic genotypes of *Nostoc* establish well-defined symbioses with a plethora of lichen-forming fungi [1,2], some thalloid bryophytes [3,4], all cycads [5,6], and species of the angiosperm genus *Gunnera* [7–9]. Symbiotic *Nostoc* genotypes, or cyanobionts, can be assigned to two main groups. One clearly defined, monophyletic lineage includes *Nostoc* cyanobionts that are exclusively shared by a wide variety of lichen-forming Ascomycota that are collectively called the *Nephroma* guild. The other *Nostoc* cyanobionts represent a much more heterogeneous assemblage and do not constitute a monophyletic clade. This group includes cyanobionts of various cyanolichens, particularly *Peltigera* species, cyanobionts of plants, and many non-symbiotic *Nostoc* genotypes [10–15].

Group 1 introns are RNA enzymes that catalyze their own splicing [16–18]. The cyanobacterial group 1 tRNALeu (UAA) intron (*trnL*) interrupts the tRNA gene and is generally believed to be of ancient origin [19]. It is present in most cyanobacterial lineages as well as in the plastids of many eukaryotic algae and all green plants [20–22]. The intron is, however, missing from some modern cyanobacteria and its mosaic-like distribution has been explained by one acquisition and several subsequent losses [19].

The self-splicing ability of *trnL* is dependent on the RNA secondary structure consisting of highly conserved sequence regions [17,18]. In addition, the intron contains more variable regions, like the P6b stem-loop and P9 region in the *trnL* intron of genus *Nostoc* [21]. The considerable sequence length variation observed in the *Nostoc trnL* P6b stem-loop is mainly caused by processes other than random mutations [21,23]. The stem-structure of the stem-loop is always built upon one of two distinct repeat motifs: either the Class I repeat motif TDNGATT or the Class II motif NNTGAGT [21]. The distribution of these two motifs does not correspond precisely with the phylogeny of *Nostoc*, and a similar dichotomy of the same two repeat motifs has also been identified from *Calothrix*, another genus of Nostocalean cyanobacteria [24].

*Nostoc trnL* sequences with a Class I P6b region can be further divided into two groups on the basis of signature characters both in the P6b stem-loop and the more conserved parts of the intron [12,24]. The clade of *Nephroma* guild lichen cyanobionts all have a Class I repeat motif, AATCTTC in the P6b central loop and a characteristic 71T in the P5b stem-loop. The sequence evolution of this group of *Nephroma*-type *Nostoc* cyanobionts is fairly well understood [23]. The lichen cyanobionts with Class I P6b regions that fall outside this clade do not share the above-mentioned signature characters [12,24], and are collectively referred to as *Collema*-type *Nostoc* cyanobionts.

The phylogenetic classification of prokaryotes is largely based on the phylogeny of the small subunit ribosomal RNA, even though the 16S rRNA gene has been shown to be prone to horizontal gene transfer (discussed for example in [25,26]). The 16S rRNA gene is routinely used in constructing phylogenies also in cyanobacteria [27–30]. As the *trnL* intron is easy to amplify and shows sufficient variability especially in the P6b region, it is widely utilized for DNA based identification of symbiotic *Nostoc* genotypes. In addition, the more conserved parts of the *trnL* intron have been used to construct phylogenies, often alongside the 16S rRNA gene, but also with other markers including the genes encoding the ribulose bisphosphate carboxylase (*rbcL*), homocitrate synthase (*nifV*), and RNA polymerase (*rpoC*), and/or the internal transcribed spacer (ITS) between the 16S and 23S rRNA genes (e.g. [31,32]).

In this study we clarify the evolution of the *trnL* P6b region in lichen-symbiotic *Nostoc*. We compare the congruence of the *trnL* intron, 16S rRNA gene, *rbcLX*, *nifV1*, and *rpoC2*, and evaluate the aptitude of Saguaro, a recently published application for the genome-wide detection of distinct local relationship patterns [33], to detect the evolutionary patterns between and within genetic markers.

## Material and Methods

### Taxon sampling and molecular markers

The data was compiled to give an extensive representation of the variation of the 16S rRNA gene and *trnL* markers in lichen-symbiotic *Nostoc*. Our new sequence data represent lichen cyanobionts of 52 lichen taxa representing 11 different genera. This data was complemented with data acquired from the NCBI GenBank and the full data set contained 78 lichen symbiotic *Nostoc* genotypes. Three lichen-symbiotic genotypes of *Rhizonema* [34,35] were used as outgroup in the phylogenetic analyses (S1 Table).

Either fresh or dried lichen specimens were used for DNA extractions, and two genomic regions were sequenced for all specimens: the approximately 1,700 nt 16S rRNA gene and the 270–372 nt *trnL* intron. DNA isolation, amplification and sequencing of the 16S rRNA gene and *trnL* intron were performed following Fedrowitz *et al*. [15,36]. Sequences were edited with Bioedit v7.0.9.0 [37] or PhyDE v1.0 [38] and primer sequences were eliminated. All sequences were submitted to GenBank, and accession numbers are listed in S1 Table together with corresponding voucher information. The collection permits for the fresh material were issued by Special Forest Products Coordinator John Poet, USDA Forest Service, and Medford District Manager Mary Smelcer, U.S. Department of the Interior Bureau of Land Management, for Oregon, and Forest Officer Julie K. Nelson, USDA Forest Service, and Natural Heritage Manager Carol Pehl, California Department of Parks and Recreation, for California localities.

In addition to the 16S rRNA gene–*trnL* sequence set, we included another data set comprised of four gene regions sequenced from lichen-symbiotic *Nostoc* [31]: *rbcLX* consisting of the *rbcX* and the intergenic spacer between *rbcL* and *rbcX*, *nifV1*, *rpoC2*, and *trnL*. All genotypes with data from all four genes in GenBank were included. After removing the P6b region from *trnL* sequences and the 74–158 nt long intron present in three *rbcLX* genotypes, all four genes were compiled into a single data set and duplicates removed resulting a data set of 21 unique *Nostoc* genotypes (S2 Table).

### Alignment and phylogenetic analyses

Alignment of the sequence data was performed manually in PhyDE v1.0, based on the criteria laid out by Kelchner [39]. As the P6b region does not always reflect true phylogenetic relationships in broad taxonomic studies [23,24,40,41], this region was omitted from the phylogenetic analyses.

Bayesian analyses were performed with MrBayes v3.2.1 [42] first for the 16S rRNA gene and *trnL* separately, and then for the combined data set. To allow possible deviating substitution models for the different regions, the data set was divided in a partition of subsets according to the markers. The best fitting nucleotide substitution models were selected by jModelTest using Akaike and Bayesian information criteria [43]. For the 16S rRNA gene the General Time Reversible nucleotide substitution model [44] with Gamma distributed rate variation among sites and proportion of Invariable sites (GTR+Γ+I) was applied. For the *trnL* the GTR+Γ model was used. The *a priori* probabilities supplied were those specified in the default settings of the program. Posterior probability distributions of trees were calculated using the Metropolis-coupled Markov chain Monte Carlo (MCMCMC) method and the search strategies suggested by Huelsenbeck *et al*. [45,46]. Four runs with four chains (1 x 10^7^ generations each) were run simultaneously, with the temperature parameter set to 0.1 but otherwise with default settings. Chains were sampled every 1,000 generations and calculations of the consensus tree and of the posterior probability of clades were performed based upon the trees sampled after the generation 2,500,000. The convergence of the chains was confirmed with Tracer v1.5 [47].

Maximum likelihood (ML) analyses were performed with Garli v2.0 [48]. For the 16S rRNA gene–*trnL* data set ML analyses with 500 replicates were run with default settings. Bootstrap (BS) analysis was performed with 10,000 BS replicates. All analyses of the 16S rRNA gene–*trnL* data set with MrBayes and Garli were performed on Bioportal [49].

For the combined data set of *rbcLX*, *nifV1*, *rpoC2*, and *trnL*, the ML analyses were performed on CIPRES Science Gateway [50]. The data set was divided in a partition of subsets according to the boundaries of the combined genes. Two times eight search replicates were run using GTR model for all regions. The BS analyses were performed with 100 BS replicates. The results of the BS analyses were summarized using SumTrees v3.3.1 [51] and all the trees were visualized using TreeGraph2 [52] and edited manually.

### Ancestral character state reconstruction

The evolutionary history of the Class II and *Collema*-type Class I *trnL* P6b regions was reconstructed by determining the posterior probability for their occurrence in the ancestral species. The Markov chain model implemented in BayesTraits v1.0 [53] was used to estimate the posterior probability distributions of ancestral states at selected nodes of the Bayesian 16S rRNA gene–*trnL* tree. For the analysis the presence of Class II *trnL* p6b region was set as one state of character and Class I (*Collema*- and *Nephroma*-types) as another. A set of 600 best trees found by MrBayes was used and the three outgroup species were pruned from the trees using R v3.0.0 package APE [54,55]. The rate at which parameters get changed (‘ratedev’) was tested and set to 2.75 so that the acceptance rate of the proposed changes ranged between 20 and 40%. A uniform distribution with a range of 0–100 was used as prior. Rate coefficients and ancestral character states were sampled every 1,000 generations to ensure independence from successive samplings. The chain was run for 5,050,000 generations. In order to circumvent issues associated with the fact that not all of the trees necessarily contain the internal nodes of interest, reconstructions were performed using a ‘most recent common ancestor’ approach. The approach identifies the most recent common ancestor to a group of species and reconstructs the state at the node for each tree and then combines this information across trees [53].

### Haplotype networks and secondary reconstruction of the *trnL* P6b regions

The haplotype network was calculated with Network v4.6.0.0 [56] using median-joining and the resulting network was edited manually. The network was calculated for the *Collema*-type *trnL* P6b regions to illustrate the sequence similarity of the regions. The constructed network and the results of the phylogenetic analysis were used to formulate a hypothesis about the sequence evolution in the *Collema*-type P6b regions.

Secondary structure reconstructions of the *Collema*-type and Class II *trnL* P6b regions were calculated from the RNA equivalent of a DNA sequence with NUPACK [57–59] using the NUPACK web server [60]. The folding temperature was set in 20°C.

### Saguaro analyses

To further elucidate the congruence between and within different markers we used the software Saguaro [33]. By combining Hidden Markov Model (HMM) with a Self-Organising Map (SOM), Saguaro compares sections within the alignment, detects sections with separate phylogenies, and gives an estimation of relations of the separate sections. The analysis results provide ‘cacti’, which are distance matrices representing a topology supported by one or several alignment segments. If the alignment includes incongruent regions, in other words alignment segments with sufficient support for dissimilar topologies, the analysis results several cacti. In addition to cacti, the analysis produces a topology reflecting the similarity of the different cacti. Saguaro analyses were run for forty iterations with default settings except for skipping positions in which all calls are identical. Separate analyses were run for the 16SrRNA gene–*trnL* and the *rbcLX*–*nifV1*–*rpoC2*–*trnL* data sets.

Based on the Saguaro analyses, additional phylogenetic analyses were performed. The 16S rRNA gene and *trnL* segments supporting the two most supported Saguaro topologies (cacti 39 and 2) were analyzed by Bayesian methods first separately and then together. For the combined *rbcLX*–*nifV1*–*rpoC2*–*trnL* data set, segments supporting all three supported Saguaro topologies (cacti 1, 3, and 34) were analyzed separately using ML. The analyses were done in the same manner as described for the corresponding complete gene regions, but two times 50 ML search replicates were run for the Saguaro segments of the *rbcLX*–*nifV1*–*rpoC2*–*trnL* data set.

### Recombination tests

To detect potentially conflicting phylogenetic signals in the gene regions, parsimony split analyses using SplitsTree4 [61] were performed. As implemented in the program, a Pairwise Homoplasy Index (PHI) [62] was calculated to test for the role of past recombination in generating allelic variation. In addition, the Genetic Algorithm Recombination Detection (GARD) approach with β-Γ rate distribution was used to further evaluate recombination and identify recombination breakpoints for each gene [63]. The GARD analyses were run on Datamonkey webserver (http://www.datamonkey.org/GARD) [64]. All data sets (S3 Table) were first analyzed with the model selection tool to define the correct nucleotide substitution bias model for the GARD analysis. GARD tests for the 16S rRNA gene were performed with and without outgroup sequences. Because of the webserver time limit for each analysis, the 16S rRNA gene analyses stopped before reaching convergence. For this reason the analyses were also performed with reduced taxon sampling. In the end, we were able to successfully run the complete analyses with 15 sequences including an outgroup and with 16 sequences without an outgroup (S3 Table). With the reduced 16S rRNA gene data set and the complete *trnL* data set the analysis was also run with rate classes set to five. GARD test for the trnL gene was performed for all sequences together without the P6b region and in addition including the P6b region for four different sequence sets: sequences with Class II repeat motifs, sequences with *Collema*-type Class I repeat motifs, sequences with *Nephroma*-type Class I repeat motifs, and all sequences with Class I, *Nephroma*- or *Collema*-type repeat motifs. For the sequences from the *rbcLX*–*nifV1*–*rpoC2*–*trnL* data set the recombination tests were performed separately for four different regions: for *rbcLX* with and without the long insert in the intergenic spacer region, for *nifV1*, and for *rpoC*2.

## Results

### The alignment and phylogenetic analyses of the genetic regions

The length of the 16S rRNA gene–*trnL* alignment was 1680 nt of which 1477 nt were 16S rRNA gene and 203 nt *trnL* (P6b region excluded). The alignment included 148 variable positions in the 16S rRNA gene and 41 in the *trnL* region. The full sequence set of 78 *Nostoc* genotypes contained 61 variable 16S rRNA gene and 71 (P6b region included) *trnL* sequences. The differences include both single nucleotide polymorphism and length variation. Of the 78 *Nostoc trnL* sequences included in this study, 34 had a *Peltigera* Class II type P6b region (33 different genotypes), 22 had a *Collema* Class I type P6b region (20 different genotypes), and 22 had a *Nephroma* Class I type P6b region (18 different genotypes).

**Fig 1:**
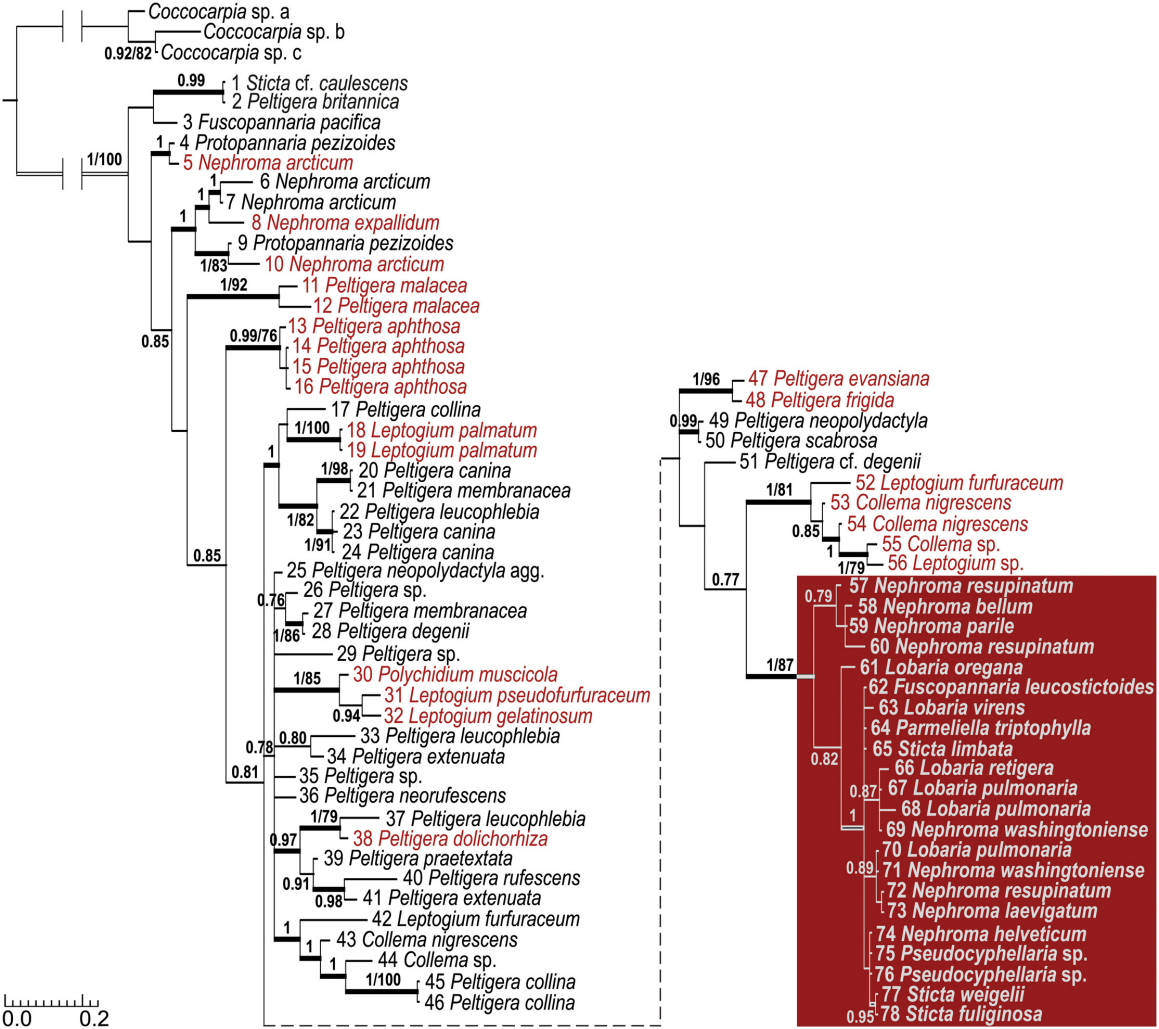
Phylogenetic relationships of the selected lichen cyanobionts. The phylogeny is based on 16S rRNA gene and *trnL* intron sequences (P6b region omitted). The posterior probability values (PP) equal or greater than 0.75 and bootstrap values (BS) equal or greater than 75 are shown on branches (just PP or PP/BS). Thick branches have PP equal or greater than 0.95. Specimens having a Class II-type *trnL* P6b region are marked black, a *Collema*-type Class I P6b region red, and a *Nephroma*-type Class I P6b region white on red background.

The separate Bayesian analyses of the 16S rRNA gene and *trnL* sequences resulted in trees with relatively poor resolution (S1 Fig). Visual comparison of the resolved parts of the trees revealed very little conflict between the two loci on the inspection threshold of Posterior Probability (PP) 0.8. In addition, when the regions were combined for Bayesian analysis, it resulted in a tree with higher resolution and more supported clades than either of the two individual analyses. This suggested that the phylogenetic signals from the 16S rRNA gene and *trnL* are not in conflict, and the combined data set could be subjected for further analyses. The Bayesian analysis of the combined 16S rRNA gene–*trnL* data set identified altogether 30 well supported (PP ≥ 0.95) clades among the lichen-symbiotic *Nostoc* (Fig 1). The ML analysis found the best tree with identical topologies 22 times, and the topology was mostly congruent with the final tree obtained by Bayesian analysis. The crown group of the Bayesian tree consisted of the *Nostoc* cyanobionts of *Nephroma* guild lichens that have *Nephroma*-type Class I *trnL* P6b regions (PP 1 / BS 87). Conversely, the *Nostoc* cyanobionts with *Collema*-type P6b regions are dispersed over ten different clades, and frequently occur mixed with *Nostoc* genotypes with Class II P6b regions.

In the ML analyses of the *rbcLX*–*nifV1*–*rpoC2*–*trnL* data set, the same best tree topology was found in eight of the altogether 16 runs (S2A Fig). The analysis found seven well supported (BS ≥ 90) groups, of which one was formed by the two included *Nephroma*-type *Nostoc* cyanobionts (BS 100). The 14 *Nostoc* genotypes with *Collema-*type *trnL* P6b regions were divided into two strongly supported groups separated by a clade including the five *Nostoc* genotypes with Class II *trnL* P6b regions.

### Ancestral character state analysis and haplotype networks

**Fig 2:**
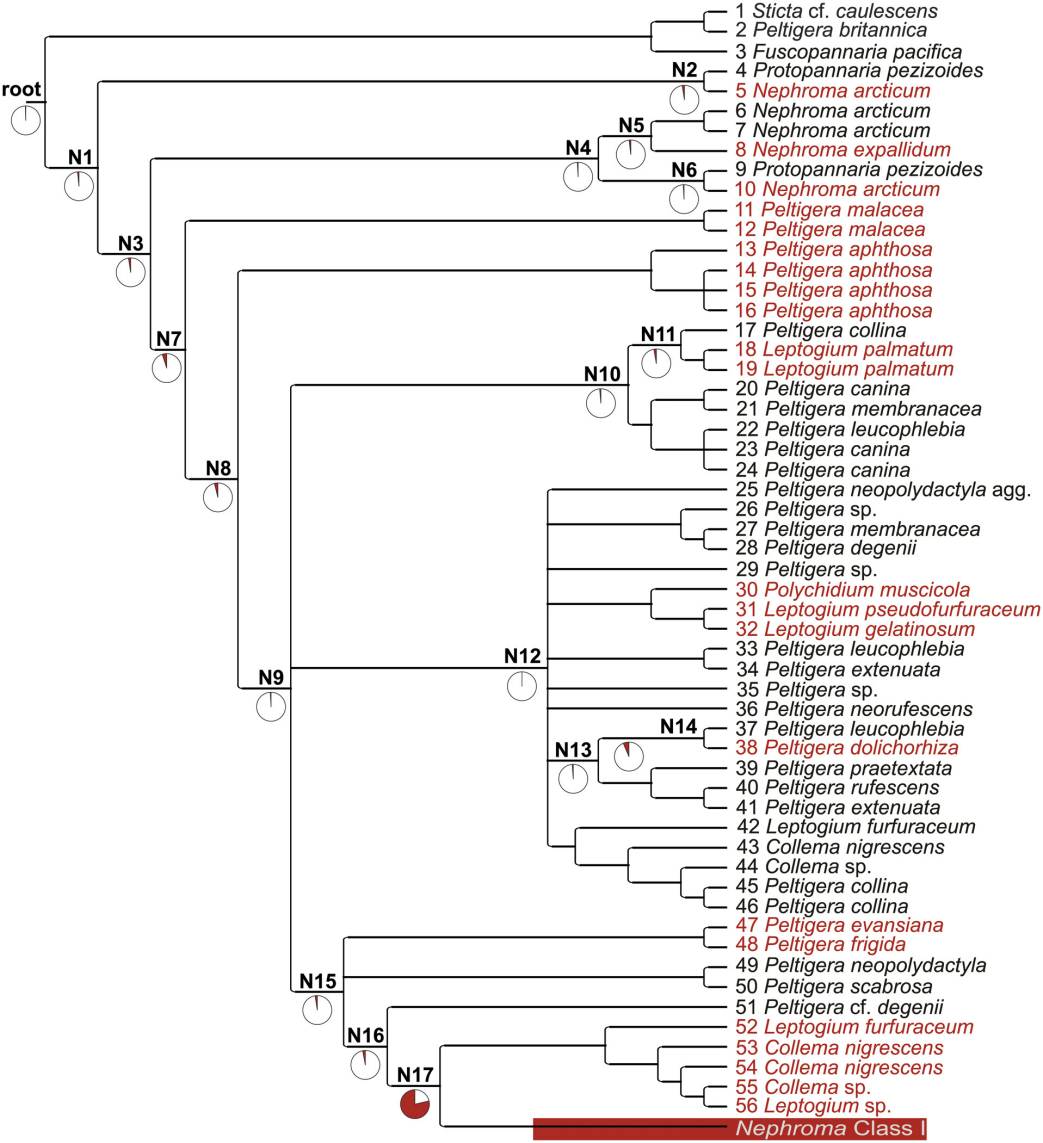
*trnL* P6b region ancestral character state reconstruction. A cladogram from the bayesian analysis of the combined 16S rRNA gene–*trnL* data set with reconstructed ancestral character states for the *trnL* P6b region. Black specimens have a Class II and red specimens *Collema*-type Class I *trnL* P6b region. The pie charts show the probability of the presence of Class II (white) or Class I (*Collema* and *Nephroma* types; red) *trnL* P6b region for the ancestral nodes. The clade formed by the cyanobionts of *Nephroma* guild lichens has been collapsed to a single branch for the figure.

The ancestral character reconstruction analysis estimates that the probability of the common ancestor of the *Nostoc* clade having a Class II p6b region is more than 99% (Fig 2). The probability of the other analyzed ancestral nodes having a Class II P6b region varied from 93 to over 99%, except for the final 17th node. The 17th node is the common ancestor of the *Nephroma* guild *Nostoc* cyanobionts with *Nephroma*-type Class I P6b regions and their sister group with *Collema*-type *Nostoc* P6b regions, and it was the most probable analyzed ancestor with a Class I P6b region with a probability of 79%.

**Fig 3:**
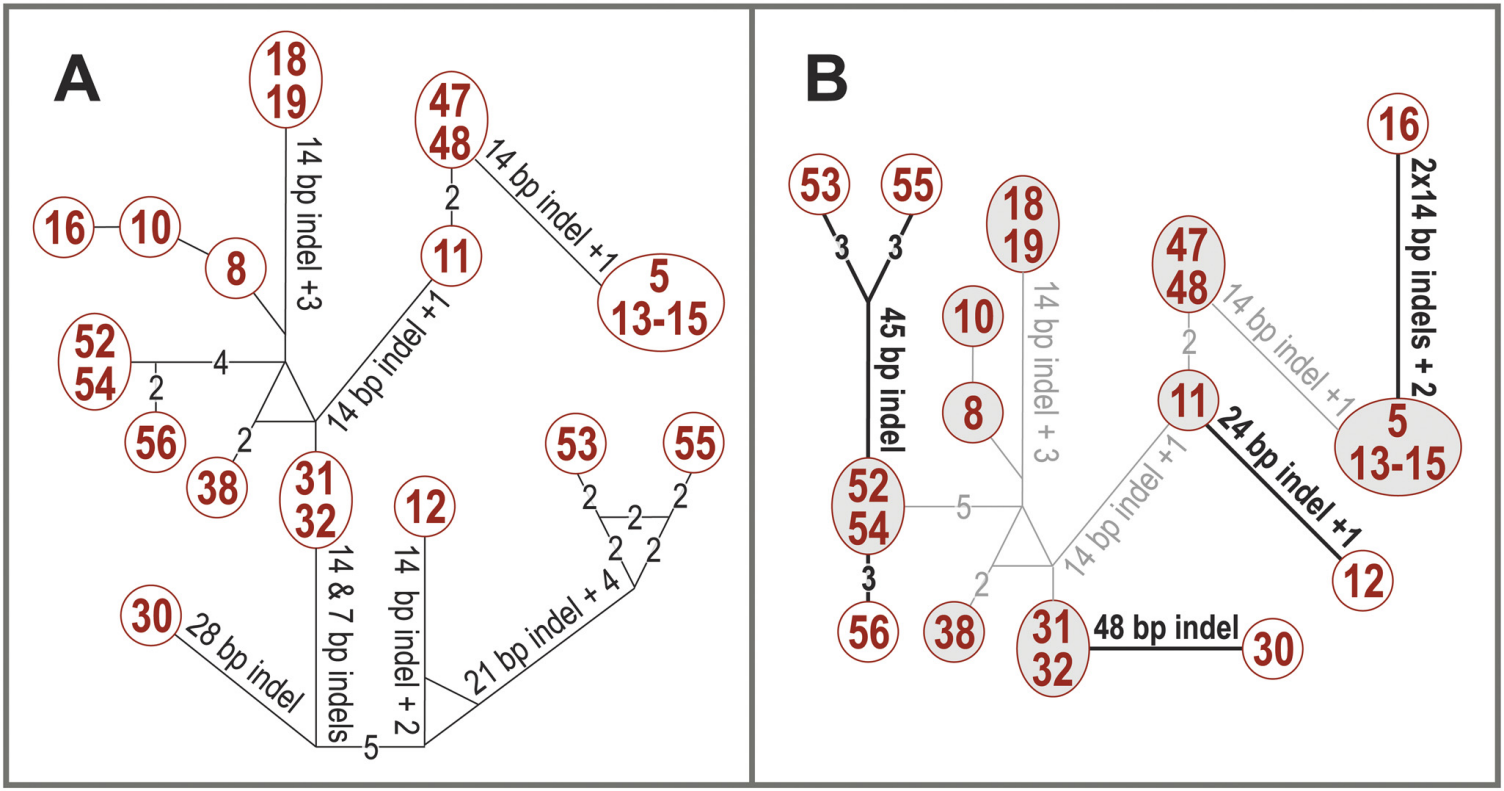
Haplotype networks showing the differences in the *Collema*-type *trnL* P6b regions. (A) Haplotype network constructed from the *Collema*-type *trnL* P6b regions with the program Network. (B) Haplotype network modified to reflect the possible evolutionary events in the *Collema*-type P6b region. One sequence from each clade present in the phylogenetic tree (Fig 1) was put together with the program Network (grey background and connecting lines). These connections reflect the overall similarity of the sequences and the different P6b types are probably results of independent adoption events. The rest of the P6b sequences were connected to their above mentioned phylogenetic relatives with the least amount of changes (black lines; each indel event or single nucleotide mutation equals one change), and the probable actual indel events and single nucleotide mutations are also marked with black.

The single nucleotide similarity based relations between the *Collema*-type *Nostoc trnL* P6b regions do not follow the phylogenetic reconstruction of the group (Fig 3A). However, if each indel event is only counted as one change and the haplotype network modified according to the obtained phylogeny, a simpler network is obtained (Fig 3B): the latter network is 15 changes shorter (altogether 40 changes) than the network constructed by the program (altogether 55 changes). The only major change seen in the latter network is represented by the long branch between *Nostoc* sequence 16 and the rest of the *Peltigera aphthosa* group *Nostoc* sequences (13–15).

### *trnL* P6b region secondary structure reconstruction analyses

The Class I P6b regions of *Collema*-type *Nostoc trnL* genotypes are more variable in length (varying from 50 to 126 nt) and both by calculated free energies of the whole P6b structure (from -53.25 to -18.70 kcal/mol) and by calculated free energy per nucleotide (from -0.426 to -0.274 kcal/mol) than the *Nostoc trnL* genotypes with Class II P6b regions (with corresponding values of 52–83 nt, -39.44…-22.04 kcal/mol, and -0.460…-0.323 kcal/mol/nt, respectively; S4 Table). In addition to being more variable, the *Collema*-type P6b structure is also less stable when the free energy is calculated per nucleotide (average -0.35 in *Collema-*type and -0.40 in Class II).

**Fig 4:**
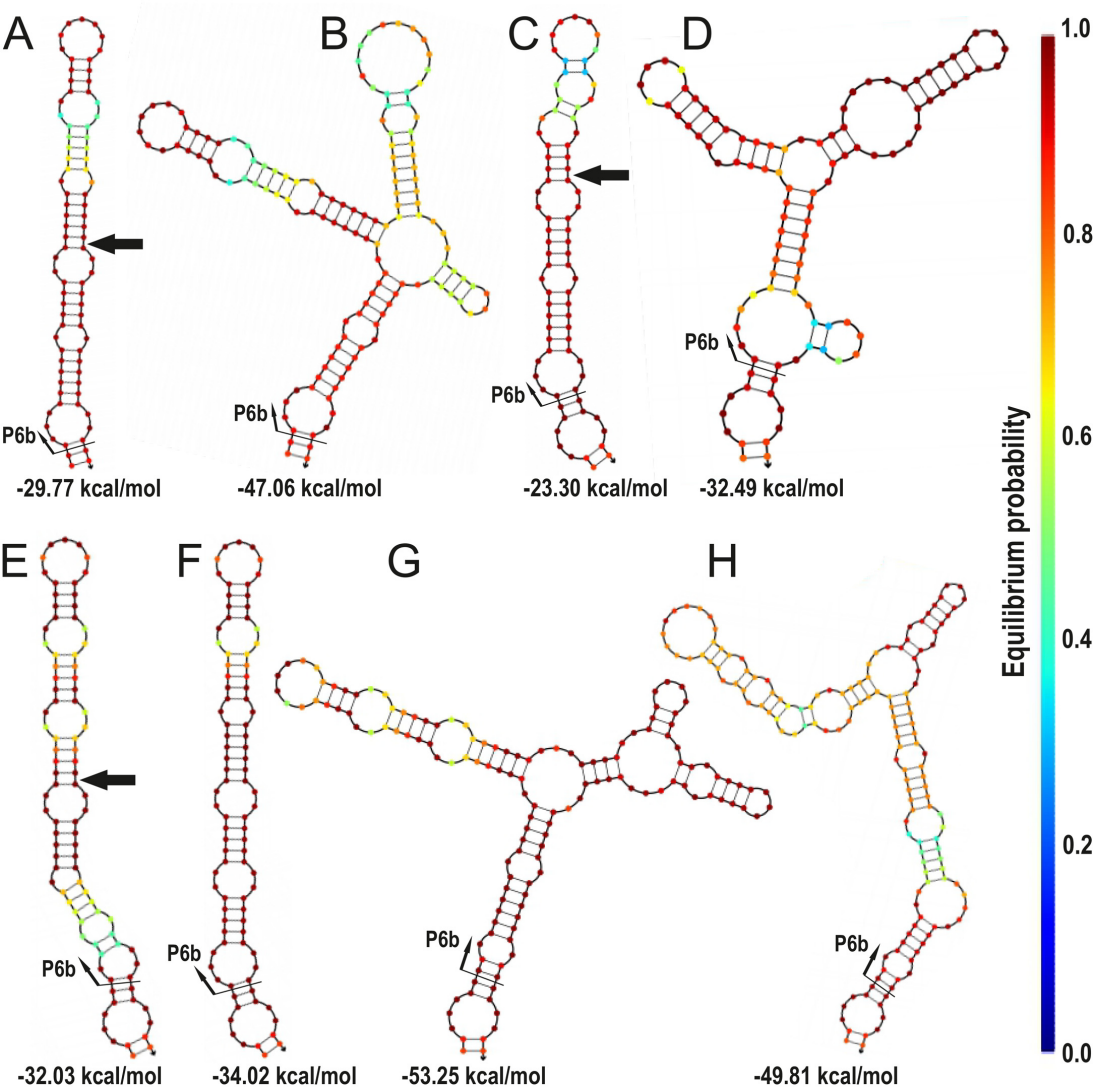
Insertions leading to side-pin structures in the secondary structure reconstruction of the *Collema*-type P6b regions. The folding of the P6b region sequences of specimens (A) 31 and 32, (B) 30, (C) 11, (D) 12, (E) 52 and 54, (F) 56, (G) 53, and (H) 55 were folded with NUPACK at 20°C. The color of each position reflects the stability of the structure, dark red being the most stable, and beneath is the calculated Gibbs free energy (ΔG) for each structure. Black arrow in (A), (C), and (E) show the position of the insertion leading to the structures shown in (B), (D), (G), and (H), respectively.

**Fig 5:**
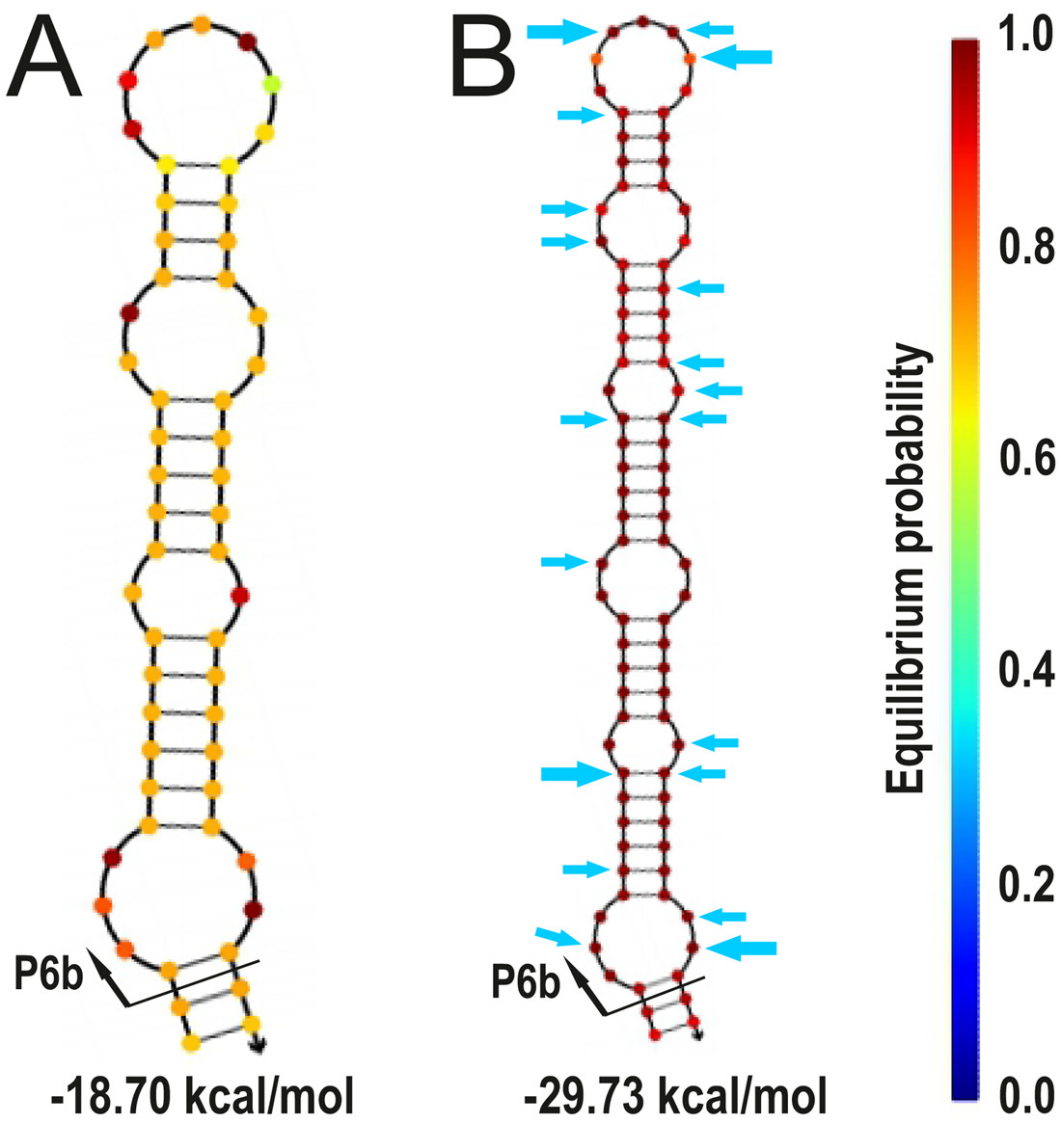
Secondary structure reconstruction of the *Collema*-type P6b regions 5 and 13–15 and 16. The folding of the P6b region sequences of specimens (A) 5 and 13–15, and (B) 16 were folded with NUPACK at 20°C. Beneath each structure is the calculated Gibbs free energy (ΔG). Color of each position reflects the stability of the structure, dark red being the most stable. The blue arrows in (B) show the single nucleotide polymorphism present in the analyzed data (bigger arrow indicates three possible character states and the smaller two).

If the phylogenetic groups that have *Collema*-type P6b regions are inspected individually, adding an insertion normally lowers the overall free energy, also when calculated per nucleotide, hence stabilizing the structure (Fig 4, S4 Table). Also, most of the indel events happen in the same position of the folded structure causing an emergence of a side stem-loop structure(s) (Fig 4). Only in the *Peltigera aphthosa* group (sequences 13–16) the indel events have happened in different parts of the structure and included two separate 14 nt insertions of the basic Class I repeat motif, this resulting in a long stem-structure without side stem-loops (Fig 5).

**Fig 6:**
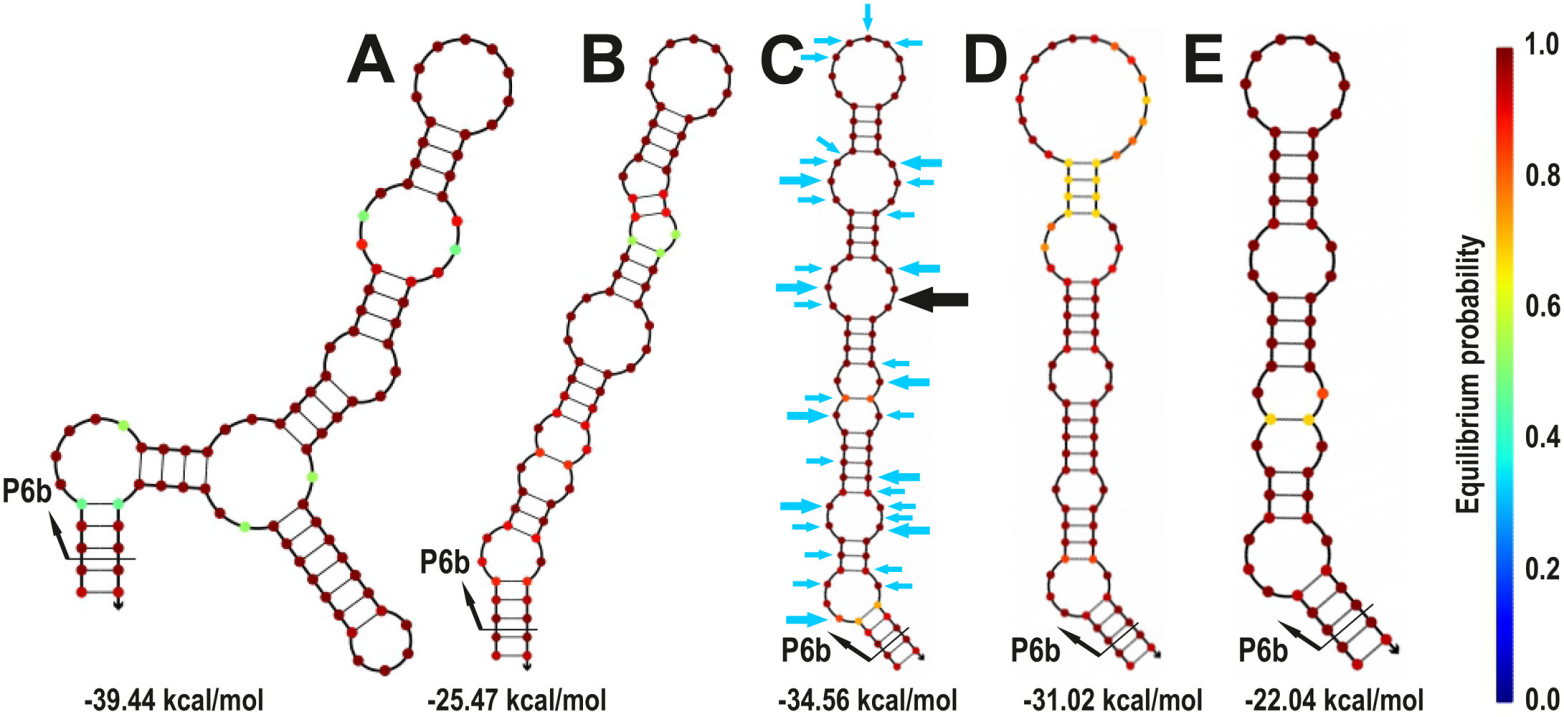
Secondary structure reconstruction of the Class II P6b regions. The folding of the P6b region sequences of specimens (A) 2, (B) 17, (C) 23, (D) 25, and (E) 27 were folded with NUPACK at 20°C. Beneath each structure is the calculated Gibbs free energy (ΔG). Color of each position reflects the stability of the structure, dark red being the most stable. The blue arrows in (C) show the single nucleotide polymorphism present in the analyzed data (bigger arrow indicates three possible character states and the smaller two) and the black arrow in (C) points the position of the insertion resulting in a side stem-loop structure shown in (A).

In Class II P6b regions only one insertion causes the formation of a side stem-loop (Fig 6A). In addition, three indel combinations involving different numbers of the basic Class II repeat motifs cause length variation in the basic stem-structure without side stem-loops (Fig 6B–6E). Two of the three indel types include events in both sides of the stem implying two separate indel events (Fig 6B and 6D), and one has only lost or gained one seven nucleotide repeat on the one side of the stem, resulting in a large head loop (Fig 6D).

When the single nucleotide differences are compared, the alignment of Class II P6b regions include altogether 33 variable single nucleotide positions (Fig 6C), while the *Collema*-type P6b regions include only 19 (Fig 5B). Of these variable positions eleven (58%) in the *Collema*-type sequences and 26 (79%) in the Class II sequences are situated in the unpairing loop regions of the structures.

### Saguaro analyses and phylogenetic analyses based on Saguaro segments

**Fig 7:**
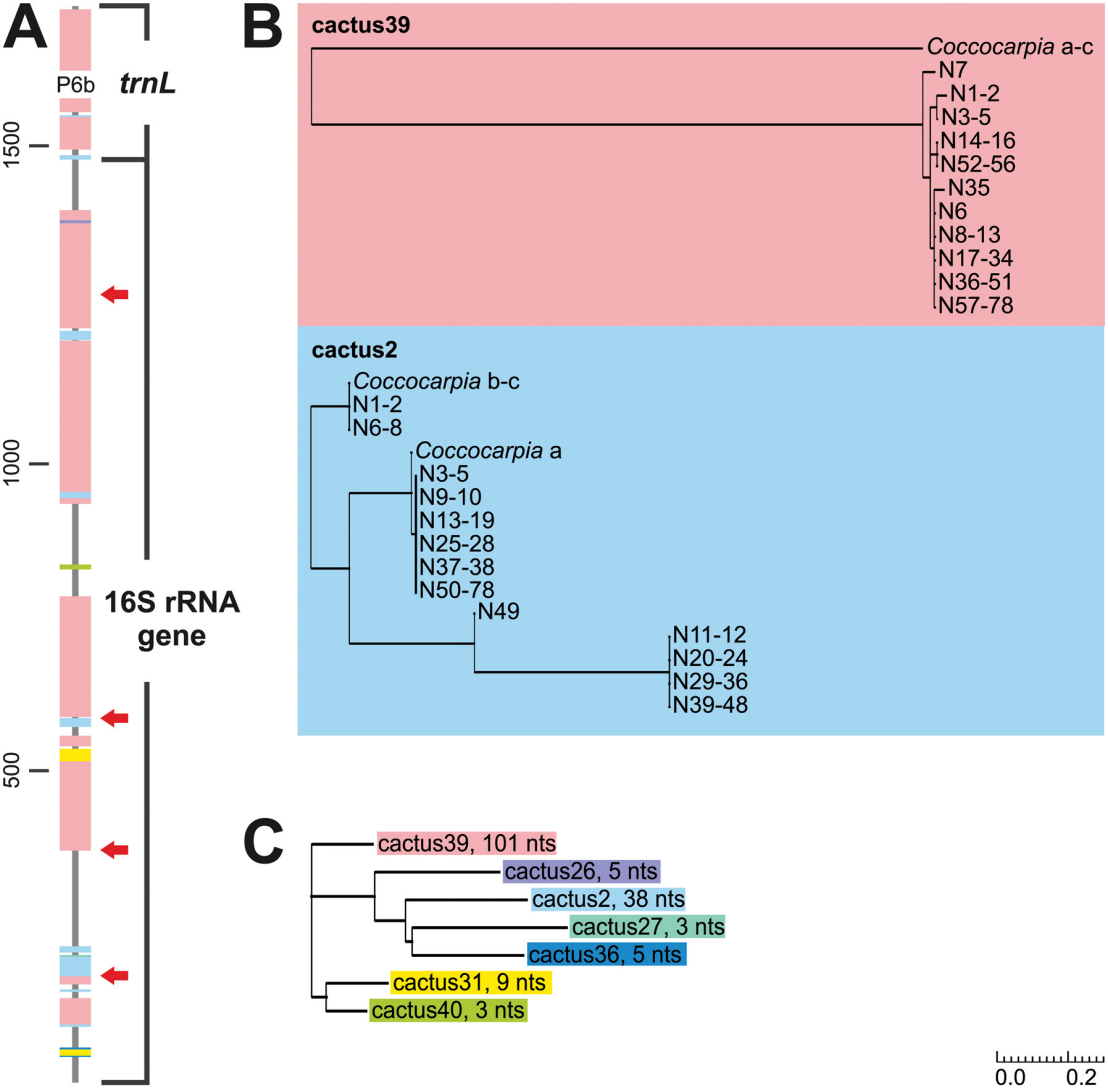
The results of the 16S rRNA gene–*trnL* Saguaro analysis. (A) Segments supporting different topologies mapped on the analyzed 16S rRNA and *trnL* genes. Each color represents a different topology (cactus). The narrower gray areas include the positions that did not support any of the filtered cacti. The red arrows point the positions of the recombination breakpoints detected in 16S rRNA gene. (B) Two the most supported topologies, cacti 39 and 2. (C) Relations between the different topologies. Following each name is the number of variable nucleotides supporting the topology.

Saguaro analysis of the 16S rRNA gene and *trnL* identified seven different cacti supported by the data set. The alignment segments supporting the cacti included 164 of the 189 variable nucleotide positions present in the alignment (Fig 7). The most supported topology, cactus 39, was supported by 11 separate alignment segments. These segments included 71 variable positions in the 16S rDNA and 30 variable positions in the *trnL* region, covering 48 and 73% of all variable nucleotide positions of the regions, respectively. Also the second most supported topology, cactus 2, was supported by alignment segments present in both 16S rRNA gene and *trnL* regions, including altogether 38 variable positions distributed among nine separate segments. The remaining five cacti were all supported by less than ten variable nucleotide positions, and all these within the 16S rRNA gene.

The topology reflecting the topological similarities of the detected cacti divided the seven cacti into three different clades (Fig 7C). One clade was formed by the most supported topology cactus 39 alone, the second by the second most supported topology cactus 2 together with three other cacti, and the third by two cacti supported by altogether 12 variable positions. The most supported topologies cacti 39 and 2 have several differences (Fig 7B): cactus 39 mainly distinguishes the *Rhizonema* sequences (outgroup) from the *Nostoc* sequences (ingroup) while cactus 2 divides all the sequences into four groups, then also mixing in- and outgroup sequences.

The Bayesian trees constructed separately of the alignment segments supporting topologies cacti 39 and 2 included relatively few supported groups. They did not show drastic incongruence between the different regions, but only relatively minor differences in the placements of single taxa (S3A and S3B Fig). Also the Bayesian trees show that the first topology mainly supports the division between out- and ingroup taxa and the second shows more variation within the ingroup. When the segments supporting these two topologies were analyzed together, Bayesian inference produced a tree with 27 strongly supported (PP ≥ 0.95) clades (S3C Fig). The tree includes some topological differences in comparison to the tree constructed of the whole gene regions (Fig 1), the most essential being the joining of the *Peltigera aphthosa* cyanobiont group (13–16) together with the gelatinous lichen cyanobionts (52–56) to form a strongly supported (PP = 0.99) sister clade to the *Nephroma* guild cyanobionts.

**Fig 8:**
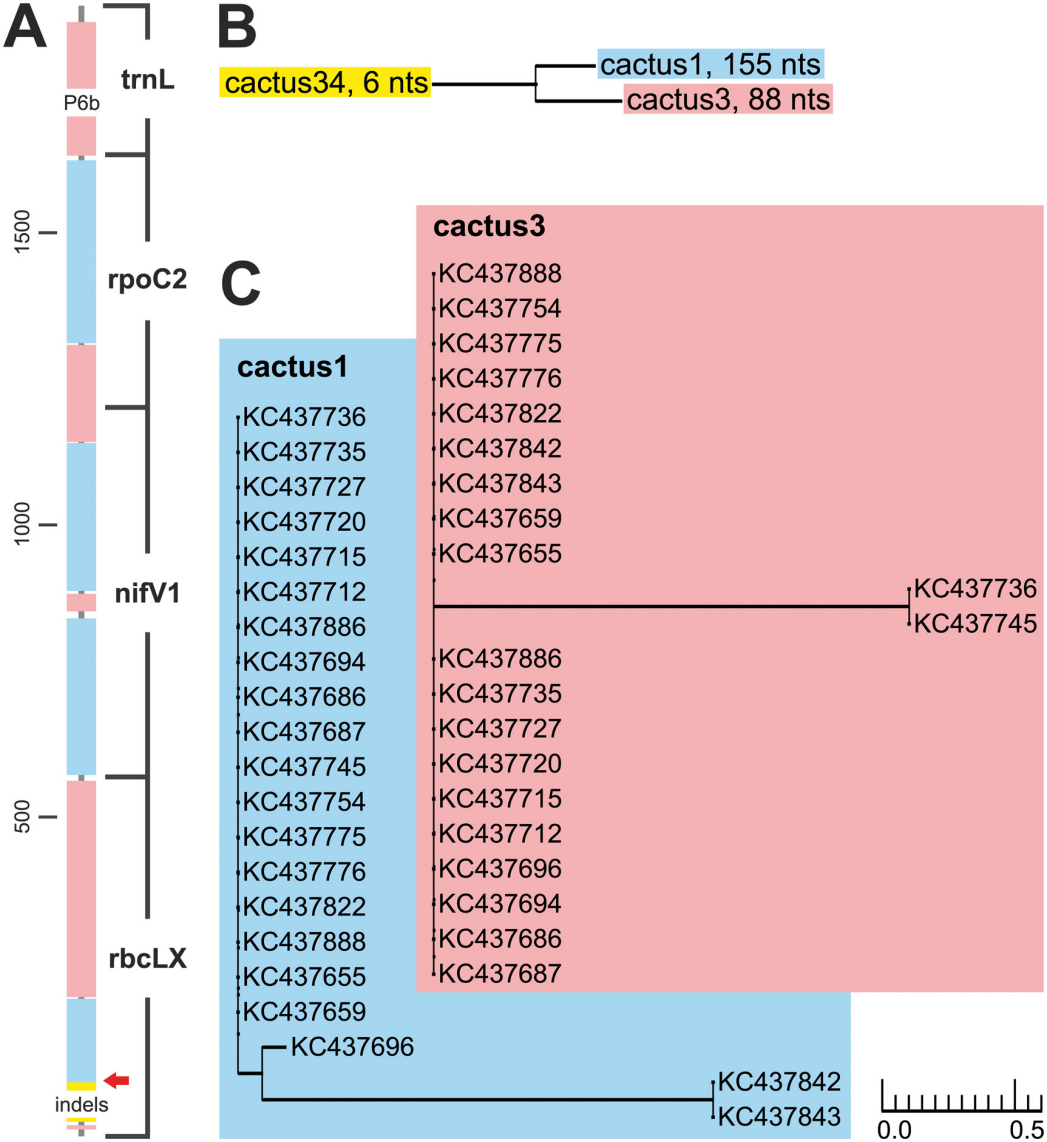
The results of the *rbcLX*–*nifV1*–*rpoC2*–*trnL* Saguaro analysis. (A) Segments supporting different topologies mapped on the analyzed genes. Each color represents a different topology (cactus). The narrower gray areas include the positions that did not support any of the filtered cacti. The red arrow points the position of the recombination breakpoint detected in *rbcLX*. (B) Relations between the different topologies. Following each name is the number of variable nucleotides supporting the topology. (C) The two most supported topologies.

In the Saguaro analysis of the *rbcLX*–*nifV1*–*rpoC2*–*trnL* data set, three separate supported topologies were detected, of which two, cacti 1 and 3, were by far the most strongly supported (Fig 8). The third topology, cactus 34, was only supported by six variable positions in the intergenic spacer region of the *rbcLX*. The topologies produced by Saguaro did not show any conflict between the most supported cacti 1 and 3, but also very little resolution. When separate ML trees were constructed from the segments supporting the different topologies, all three trees showed some topological incongruence between the segments supporting different cacti and noticeably more resolution than the topologies provided by Saguaro (S2B–S2D Fig).

### Recombination analyses

For the *trnL* sequences, the PHI test did not find statistically significant evidence for recombination (S3 Table). Recombination was neither detected from the conserved parts of the *trnL* when all the sequences were tested together with the outgroup taxa nor when the P6b region was included in different sets of sequences.

With the 16S rRNA gene, the PHI test indicated a significant degree of recombination (p-value 0.0 with and without outgroup; S3 Table). Furthermore, the GARD approach confirmed the existence of significant recombination in 16S rRNA gene as several recombination breakpoints with significant topological incongruence between the segments were found (S3 Table). Analysis of a reduced data set of 17 sequences detected four recombination breakpoints even though the analysis did not reach convergence because of the server time limit (Fig 7). It is possible that if the analysis had continued, more breakpoints would have been detected and their exact positions might have changed.

The PHI test detected recombination also from the *rbcLX* (p-value 3.7E-5 with the insert and 2.0E-4 without), *nifV1* (p-value 5.5E-4), and *rpoC2* (p-value 0.04; S3 Table). However, GARD analysis did not detect recombination breakpoints from any other region than *rbcLX* when analyzed with the insertion (S3 Table; Fig 8).

## Discussion

### The evolution of the *trnL* P6b region in lichen-symbiotic *Nostoc*

Visualization of the distribution of the different *trnL* P6b region types in lichen-symbiotic *Nostoc* shows that the Class I P6b regions mainly occur in scattered groups of a few genotypes, with the exception of the large crown group of the phylogenetic tree (taxa 52–78, Fig 1). The crown group is formed by two well supported sister clades of *Nostoc* cyanobionts, which all harbour Class I *trnL* P6b regions. Based on the phylogeny, the lichen cyanobionts with *Nephroma*-type Class I *trnL* P6b regions clearly represent their own monophyletic lineage, as has been suggested by the results of several previous studies (e.g. [8,24,31,65,66]). The results also confirm that the *Nephroma* and *Collema*-type Class I *trnL* sequences can reliably be distinguished based on the characteristic sequence patterns mentioned in the introduction. However, the defining difference in the P6b central loop sequence, introduced as AATCTTC for the *Nephroma*-type, seems to be only the second character. In the light of the new results, the *Nephroma*-type loop varies as A**A**(T/-)TCTTY and *Collema*-type loop as W**G**(-)TBTWH.

The results of the ancestral character reconstruction of the Class I and II *trnL* P6b regions show that the scattered distribution of the *Collema* type Class I P6b regions is a consequence of several independent replacements of the Class II P6b region with a Class I region (Fig 2). Also, even though the ML tree constructed of the *rbcLX*, *nifV1*, *rpoC2*, and *trnL* gene regions is considerably smaller and lacks resolution in some parts, it shows a well supported division of the *Nostoc* genotypes with *Collema*-type *trnL* P6b regions, pointing towards the independent origin of the regions (S2A Fig). The ancestral character state analysis also suggests that the replacement has always been unidirectional. Since the *Collema* type Class I P6b region has repeatedly replaced the Class II type P6b region in different *Nostoc* lineages, and the *Nephroma* type Class I P6b regions are not found outside the monophyletic group of *Nephroma* guild *Nostoc* cyanobionts, the evolutionary changes from a *Collema* type P6b region to a *Nephroma* type P6b region appears to only have occurred once.

The repeated unidirectional replacements of Class II type P6b regions with Class I type P6b regions suggest that some structural features of Class II P6b regions increase the likelihood of such substitutions. This structural tendency may be reflected in the above average free energies of the Class II P6b secondary structures in several phylogenetic groups with *Collema-type* P6b regions (genotypes 3, 4, 6, 7, 9, and 17; S4 Table). In addition to the replacements of Class II P6b region with Class I, also replacements of one *Collema*-type P6b region with another are probable: The most parsimonious explanation for the two different *Collema*-type P6b regions present in the monophyletic *Peltigera aphthosa* group (13–16) is the replacement of the entire P6b region, and not by several indel and single nucleotide mutation events within the region. If the phylogenetic reconstruction based only on the more generally supported segments of the 16S rRNA gene–*trnL* alignment is believed to be more trustworthy (S3 Fig), at least three independent replacements have occurred within the group including cyanobionts 13–16 and 52–56, most probably between different *Collema*-type P6b regions. Thus, if mobile elements were to exist in the P6b regions of lichen-symbiotic *Nostoc* genotypes, they should most likely be found from *Collema*-type sequences.

While the nucleotide sequence of the P6b stem-loop in Class II and Class I types is very different the secondary structure is quite similar. The most important feature defining the similar structure are the two seven nucleotide repeat motifs that ensure the correct pairing and sufficient stability. While the evolutionary origins of the complementary repeat classes are not known, the presence of only one of the two repeat motifs in *Nostoc* genotypes suggests that both motifs can fulfil the same functional role. From this perspective, it is interesting that the insertions causing the side stem-loop structure tend to occur in the same position in both *Collema*-type Class I and in Class II P6b regions. The secondary structure reconstructions of the different P6b regions show, that Class II P6b regions are generally more stable and include less length variation than the *Collema*-type P6b regions. The phylogenetic reconstruction also suggests, that within lineages with *Collema*-type *trnL* P6b regions, the number of repeats is a conserved character inherited from the common ancestral P6b region of each phylogenetic group. As a whole, the Class II P6b regions include much more single nucleotide variation than the *Collema*-type P6b regions and of this variation the major part is found in the unpaired regions of the stem-loop structure, minimizing its effect on structural stability. According to the neutral theory of molecular evolution [67], the mutations in the unpaired loop regions could perhaps be used as relative molecular clocks, providing independent evidence for the ancestry of Class II P6b regions. It is also feasible that the ancestral Class II P6b structures have evolved to relatively stable states through time, while the derived *Collema*-type P6b regions are still in the process of balancing between the stabilizing benefits of long side stem-loop insertions and the costs of maintaining such structures.

### Saguaro and congruence of the markers

We tested the suitability of the program Saguaro [33] to detect evolutionary patterns between and within genetic markers. In both separate data sets, Saguaro detected that all tested markers supported more than one incongruent topologies. The results were confirmed by other analyses: Recombination breakpoints were detected from the 16S rRNA gene and from the *rbcLX*. The Bayesian and ML trees constructed from the alignment segments supporting each topology also indeed proved to be incongruent, even though they partly differed from the cacti produced by Saguaro using a fast neighbour-joining method.

The recombination breakpoints detected in the 16S rRNA gene do not separate the segments supporting the two most supported topologies cactus 39 and cactus 2, but are situated between the segments supporting other topologies. The segments situated closely together in the beginning of 16S rRNA gene supporting cacti 31 and 36, the only segment supporting cactus 27, the second segment supporting cactus 31, the only segment supporting cactus 40, and the only segment supporting cactus 26 are all separated by recombination breakpoints. On the other hand, almost all such parts include segments supporting both cactus 39 and cactus 2. This implies that there may not be much incongruence between the two topologies. However, it must be kept in mind that as the GARD analysis for the whole 16S rRNA gene sequence set could not finish, the gene region may potentially include even more recombination breakpoints. The position of the detected *rbcLX* breakpoint almost coincides with the end of the only segment supporting topology cactus 34 in the Saguaro analysis and it is in the close proximity of the end of the intergenic spacer. There are also other reports of recombination from this position in *Nostoc* [28].

Generally, incongruence was mostly detected within genetic markers rather than between the different genetic regions. The conserved parts of the *trnL* sequences did not include phylogenetic signals that were not present in the other tested markers, 16S rRNA gene, *rbcLX*, *nifV1*, and *rpoC2*. Also, no recombination was detected from the *trnL*, even when different combinations of sequences with the P6b region were analyzed. However, the *trnL* gene region is very short and this may hamper the detection of conflicting signals. 16S rRNA gene, on the other hand, included segments supporting several incongruent topologies separated also by recombination breakpoints. Most of the incongruence seems to concentrate in short alignment segments including only a few variable nucleotides, and the vast majority of the variation present is in congruence with the conserved parts of the *trnL*. Previously, when the congruence of 16S rRNA gene and other genetic markers have been evaluated, *rpoC1*, *hetR*, *rbcLX* have been found to be incongruent with 16S rRNA gene [28]. However, the comparison was made based on gene tree topologies, and no further analysis of the reason of the incongruence was made. Here, *rbcLX*, *nifV1*, and *rpoC2* were mostly mutually congruent and also congruent with *trnL*. Also these markers included segments supporting two different topologies, but such segments were present in all three markers. Only the intergenic spacer in the beginning of *rbcLX* supported a topology not supported by other parts of the gene regions. It was also separated by a recombination breakpoint and perhaps should therefore be omitted from phylogenetic analyses.

The 16S rRNA gene has been widely used in constructing cyanobacterial phylogenies. However, in our study of lichen-symbiotic *Nostoc* the 16S rRNA gene data alone could not produce a well-supported and resolved phylogenetic tree. The phylogenetic trees inferred from the 16S rRNA gene have often lacked resolution and been weakly supported also in previous studies. This ambiguity is most probably caused by the conflicting signals resulting from several recombination events, even though the conflicting alignment segments seem to be very short, comprising only a few variable nucleotides each. Many studies have found evidence of recombination events in different cyanobacterial genes [28,68–70] and the bacterial 16S rRNA gene in general has been shown to be prone to horizontal gene transfer [25,26]. The various 16S rRNA gene alignment segments supporting variable topologies and the amount of detected recombination breakpoints inside the phylogenetically relatively closely related taxa suggests that horizontal gene transfer may be relatively common in this group of cyanobacteria.

## Conclusions

Our results confirm the monophyly of the *Nephroma* guild cyanobionts with *Nephroma* type Class I P6b regions, the polyphyly of *Nostoc* cyanobionts with *Collema* type Class I P6b regions, and the clear phylogenetic distinction between these two groups. The sporadic distribution of the *trnL* P6b region types Class I and II in the lichen-symbiotic *Nostoc* is explained by several independent replacements of Class II P6b region with a Class I region. The ancestry of Class II P6b region is also supported by the total and relative amounts of the single nucleotide mutations in the more neutral, unpaired loop regions of the stem-loop secondary structure.

The 16S rRNA gene was shown to contain several recombination breakpoints and segments supporting variable phylogenetic topologies. Also other gene regions commonly used in constructing cyanobacterial phylogenies contained signs of possible horizontal gene transfer. When examining the congruence of genetic regions, trees based on the separate markers only show the possible presence of incongruence. This can lead to misjudgments, since, like in both data sets analyzed here, a lot of incongruence seems to occur within individual marker regions. In our study Saguaro proved to be a valuable tool for clarifying incongruence. In addition to recognizing conflicting signals, it helped to pinpoint the incongruent regions that need to be identified prior to phylogenetic analyses. In general, the amount of detected incongruence especially in the 16S rRNA gene and the very complex evolution of the *trnL* P6b region, emphasize the importance of such protocols, and suggests that horizontal transfer of genetic material have had a great influence in evolution also in the lichen-symbiotic *Nostoc*.

## Supporting Information

S1 Fig Separate Bayesian trees of 16S rRNA gene and *trnL*. Bayesian trees constructed of the 16S rRNA gene (left) and *trnL* (without the P6b region; right) for the visual inspection of the congruence of the markers. The nodes have been collapsed to a threshold of PP > 0.8.
(EPS)

S2 Fig Maximum likelihood (ML) trees constructed of *rbcLX*, *nifV1 rpoC2*, and *trnL* sequences. (A) ML tree constructed of all four gene regions excluding only the long introns in the beginning of *rbcLX* and the P6b region in *trnL*. Numbers in parenthesis after the accession number refer to specimens in our material having an identical *trnL* sequence (including P6b region). ML trees constructed of the alignment segments supporting topology (B) cactus 1 (the best tree was found once in hundred search replicates), (C) cactus 3 (the best tree was found four times in hundred search replicates), and (D) cactus 34 (the best tree was found once in hundred search replicates) in the Saguaro analysis. In all trees nodes with bootstrap BS support less than 50 have been collapsed. BS values equal or greater than 75 are shown on branches, and thick branches have BS equal or greater than 90. Specimens having a Class II-type *trnL* P6b region are marked black, a *Collema*-type Class I P6b region red, and a *Nephroma*-type Class I P6b region white on red background. The shown GenBank accession numbers belong to the *rbcLX* gene.
(EPS)

S3 Fig Bayesian trees constructed from 16S rRNA gene and *trnL* segments supporting different topologies in the Saguaro analysis. Bayesian trees constructed of the 16S rRNA gene and *trnL* segments supporting topologies (A) cactus 39, (B) cactus 2, and (C) cacti 39 and 2 in the 16S-*trnL* Saguaro analysis. Posterior probability (PP) values equal or over 0.75 are shown on the branches. Thick branches have PP equal or greater than 0.95. Specimens having a Class II-type *trnL* P6b region are marked black, a *Collema*-type Class I P6b region red, and a *Nephroma*-type Class I P6b region white on red background.
(EPS)

S1 Table Specimen information. List of specimens used in the study including collection information and NCBI GenBank accession numbers for the sequences. The sequences generated for this study are bolded. The collector and voucher information is presented only for newly generated sequences.
(DOCX)

S2 Table Data set of *rbcLX*, *nifV1*, *rpoC2*, and *trnL* from O'Brien *et al*. [31]. The numbering follows the numbering of the original Supporting Information S1 Table in O'Brien et al. [31]. The 'Identical *trnL*' column refers to the identical *trnL* sequences in the 16S rRNA gene–*trnL* data set.
(DOCX)

S3 Table Results of the SplitsTree and GARD recombination tests. P is the p-value of the PHI-test and RC the number of rate classes used in the GARD analysis. KH refers to Kishino Hasegawa test and * in the end of the row indicates that the GARD analysis stopped before convergence. 16S refers to the 16S rRNA gene.
(DOCX)

S4 Table *trnL* P6b region secondary structure reconstruction. ID tells the taxa in which the P6b region is present. The secondary structure reconstruction and calculations were made with a sequence where two nucleotides outside the region were included in both ends, and in the ones marked with the * six nucleotides from the beginning and seven from the end outside the P6b region were included to ensure the correct folding [17].
(DOCX)

## References

[1] Rikkinen J. Cyanolichens: an evolutionary overview In: Rai AN, Bergman B, Rasmussen U, editors. Cyanobacteria in Symbiosis. Dordrecht: Kluwer Academic Publisher; 2002 pp. 31–72.

[2] Rikkinen J. Cyanolichens. Biodivers Conserv. 2015;24: 973–993.

[3] Adams DG, Duggan PS. Cyanobacteria-bryophyte symbioses. J Exp Bot. 2008;59: 1047–1058. 10.1093/jxb/ern005 18267939

[4] Rikkinen J, Virtanen V. Genetic diversity in cyanobacterial symbionts of thalloid bryophytes. J Exp Bot. 2008;59: 1007–1011. 10.1093/jxb/ern004 18325923

[5] Costa JL, Romero ME, Lindblad P. Sequence based data supports a single *Nostoc* strain in individual coralloid roots of cycads. FEMS Microbiol Ecol. 2004;49: 481–487. 10.1016/j.femsec.2004.05.001 19712296

[6] Thajuddin N, Muralitharan G, Sundaramoorthy M, Ramamoorthy R, Ramachandran S, Akbarsha MA, et al Morphological and genetic diversity of symbiotic cyanobacteria from cycads. J Basic Microbiol. 2010;50: 254–265. 10.1002/jobm.200900343 20473963

[7] Nilsson M, Bergman B, Rasmussen U. Cyanobacterial diversity in geographically related and distant host plants of the genus *Gunnera* . Arch Microbiol. 2000;173: 97–102. 10795680 10.1007/s002039900113

[8] Bergman B. *Nostoc*-*Gunnera* symbiosis In: Rai AN, Bergman B, Rasmussen U, editors. Cyanobacteria in Symbiosis. Dordrecht: Kluwer Academic Publisher; 2002 pp. 207–232.

[9] Svenning MM, Eriksson T, Rasmussen U. Phylogeny of symbiotic cyanobacteria within the genus *Nostoc* based on 16S rDNA analyses. Arch Microbiol. 2005;183: 19–26. 15549268 10.1007/s00203-004-0740-y

[10] Rikkinen J, Oksanen I, Lohtander K. Lichen guilds share related cyanobacterial symbionts. Science 2002;297: 357 12130774 10.1126/science.1072961

[11] Rikkinen J. Ecological and evolutionary role of photobiont-mediated guilds in lichens. Symbiosis 2003;34: 443–450.

[12] Rikkinen J. Ordination analysis of tRNALeu (UAA) intron sequences from lichen-forming *Nostoc* strains and other cyanobacteria. Symb Bot Ups. 2004;34: 377–391.

[13] Rikkinen J. Molecular studies on cyanobacterial diversity in lichen symbioses. Mycokeys 2013;6: 3–32.

[14] Elvebakk A, Papaefthimiou D, Robertsen EH, Liaimer A. Phylogenetic patterns among *Nostoc* cyanobionts within bi- and tripartite lichens of the genus *Pannaria* . J Phycol. 2008;44: 1049–1059. 27041623 10.1111/j.1529-8817.2008.00556.x

[15] Fedrowitz K, Kaasalainen U, Rikkinen J. Geographic mosaic of symbiont selectivity in a genus of epiphytic cyanolichens. Ecol Evol. 2012;2: 2291–2303. 10.1002/ece3.343 23139887 PMC3488679

[16] Kruger K, Grabowski PJ, Zaug AJ, Sands J, Gottschling DE, Cech TR. Self-splicing RNA: autoexcision and autocyclization of the ribosomal RNA intervening sequence of *Tetrahymena* . Cell 1982;31: 147–157. 6297745 10.1016/0092-8674(82)90414-7

[17] Cech TR. Conserved sequences and structures of group-I introns: building an active site for RNA catalysis—a review. Gene 1988;73: 259–271. 3072259 10.1016/0378-1119(88)90492-1

[18] Cech TR, Damberger SH, Gutell RR. Representation of the secondary and tertiary structures of group I introns. Nat Struct Biol. 1994;1: 273–280. 7545072 10.1038/nsb0594-273

[19] Paquin B, Kathe SD, Nierzwicki-Bauer SA, Shub DA. Origin and evolution of group-I introns in cyanobacterial tRNA genes. J Bacteriol. 1997;179: 6798–6806. 9352932 10.1128/jb.179.21.6798-6806.1997 PMC179611

[20] Besendahl A, Qiu Y-L, Lee J, Palmer JD, Bhattacharya D. The cyanobacterial origin and vertical transmission of the plastid tRNALeu group-I-intron. Curr Genet. 2000;37: 12–23. 10672439 10.1007/s002940050002

[21] Costa JL, Paulsrud P, Lindblad P. The cyanobacterial tRNALeu (UAA) intron: evolutionary patterns in a genetic marker. Mol Biol Evol. 2002;19: 850–857. 12032241 10.1093/oxfordjournals.molbev.a004142

[22] Simon D, Fewer D, Friedl T, Bhattacharya D. Phylogeny and self-splicing ability of the plastid tRNA-Leu group I intron. J Mol Evol. 2003;57: 710–720. 14745540 10.1007/s00239-003-2533-3

[23] Olsson S, Kaasalainen U, Rikkinen J. Reconstruction of structural evolution in the *trnL* intron P6b loop of symbiotic *Nostoc* (Cyanobacteria). Curr Genet. 2012;58: 49–58. 10.1007/s00294-011-0364-0 22210193

[24] Oksanen I, Lohtander K, Sivonen K, Rikkinen J. Repeat-type distribution in *trnL* intron does not correspond with species phylogeny: comparison of the genetic markers 16S rRNA and *trnL* intron in heterocystous cyanobacteria. Int J Syst Evol Microbiol. 2004;54: 765–772. 15143022 10.1099/ijs.0.02928-0

[25] McInerney JO, Cotton JA, Pisani D. The prokaryotic tree of life: past, present… and future? Trends Ecol Evol. 2008;23: 276–281. 10.1016/j.tree.2008.01.008 18367290

[26] Kitahara K, Miyazaki K. Revisiting bacterial phylogeny: Natural and experimental evidence for horizontal gene transfer of 16S rRNA. Mob Genet Elements 2013;3: e24210 23734299 10.4161/mge.24210 PMC3661144

[27] Papaefthimiou D, Hrouzek P, Mugnai MA, Lukesova A, Turicchia S, Rasmussen U, et al Differential patterns of evolution and distribution of the symbiotic behaviour in nostocacean cyanobacteria. Int J Syst Evol Microbiol. 2008;58: 553–564. 10.1099/ijs.0.65312-0 18319454

[28] Han D, Fan Y, Hu Z. An evaluation of four phylogenetic markers in *Nostoc*: implications for cyanobacterial phylogenetic studies at the intrageneric level. Curr Microbiol. 2009;58: 170–176. 10.1007/s00284-008-9302-x 18972163

[29] Kaasalainen U, Fewer DP, Jokela J, Wahlsten M, Sivonen K, Rikkinen J. Cyanobacteria produce a high variety of hepatotoxic peptides in lichen symbiosis. Proc Natl Acad Sci U S A. 2012;109: 5886–5891. 10.1073/pnas.1200279109 22451908 PMC3326460

[30] Schirrmeister BE, de Vos JM, Antonelli A, Bagheri HC. Evolution of multicellularity coincided with increased diversification of cyanobacteria and the Great Oxidation Event. Proc Natl Acad Sci U S A. 2013;110: 1791–1796. 10.1073/pnas.1209927110 23319632 PMC3562814

[31] O’Brien HE, Miadlikowska J, Lutzoni F. Assessing population structure and host specialization in lichenized cyanobacteria. New Phytol. 2013;198: 557–566. 10.1111/nph.12165 23406441

[32] Micheli C, Cianchi R, Paperi R, Belmonte A, Pushparaj B. Antarctic Cyanobacteria Biodiversity Based on ITS and *TrnL* Sequencing and Its Ecological Implication. Open J Ecol. 2014;4: 456–467.

[33] Zamani N, Russell P, Lantz H, Hoeppner MP, Meadows JR, Vijay N, et al Unsupervised genome-wide recognition of local relationship patterns. BMC Genomics 2013;14: 347 10.1186/1471-2164-14-347 23706020 PMC3669000

[34] Lücking R, Lawrey JD, Sikaroodi M, Gillevet PM, Chaves JL, Sipman HJM, et al Do lichens domesticate photobionts like farmers domesticate crops? Evidence from a previously unrecognized lineage of filamentous cyanobacteria. Am J Bot. 2009;96: 1409–1418. 10.3732/ajb.0800258 21628288

[35] Lücking R, Barrie FR, Genney D. *Dictyonema coppinsii*, a new name for the European species known as *Dictyonema interruptum* (Basidiomycota: Agaricales: Hygrophoraceae), with a validation of its photobiont *Rhizonema* (Cyanoprokaryota: Nostocales: Rhizonemataceae). Lichenologist 2014;46: 261–267.

[36] Fedrowitz K, Kaasalainen U, Rikkinen J. Genotype variability of *Nostoc* symbionts in association with three epiphytic *Nephroma* species in a boreal forest landscape. Bryologist 2011;114: 220–230.

[37] Hall TA. BioEdit: a user-friendly biological sequence alignment editor and analysis program for Windows 95/98/NT. Nucleic Acids Symp Ser. 1999;41: 95–98.

[38] Müller K, Quandt D, Müller J, Neinhuis C. PhyDE 0.995: Phylogenetic Data Editor. 2005. Available: www.phyde.de.

[39] Kelchner SA. The evolution of non-coding chloroplast DNA and its application in plant systematics. Ann Mo Bot Gard. 2000;87: 482–498.

[40] Costa JL, Paulsrud P, Rikkinen J, Lindblad P. Genetic diversity of *Nostoc* symbionts endophytically associated with two bryophyte species. Appl Environ Microbiol. 2001;67: 4393–4396. 11526056 10.1128/AEM.67.9.4393-4396.2001 PMC93180

[41] Linke K, Hemmerich J, Lumbsch HT. Identification of *Nostoc* cyanobionts in some *Peltigera* species using a group I intron in the tRNALeu gene. Bibl Lichenol. 2003;86: 113–118.

[42] Huelsenbeck JP, Ronquist F. MrBayes: Bayesian inference of phylogenetic trees. Bioinformatics 2001;17: 754–755. 11524383 10.1093/bioinformatics/17.8.754

[43] Posada D. jModelTest: phylogenetic model averaging. Mol Biol Evol. 2008;25: 1253–1256. 10.1093/molbev/msn083 18397919

[44] Tavaré S. Some probabilistic and statistical problems in the analysis of DNA sequences In: Miura RM, editor. Some Mathematical Questions in Biology—DNA Sequence Analysis. Providence: American Mathematical Society; 1986 pp. 57–86.

[45] Huelsenbeck JP, Ronquist F, Nielsen R, Bollback JP. Bayesian inference of phylogeny and its impact on evolutionary biology. Science 2001;294: 2310–2314. 11743192 10.1126/science.1065889

[46] Huelsenbeck JP, Larget B, Miller RE, Ronquist F. Potential applications and pitfalls of bayesian inference of phylogeny. Syst Biol. 2002;51: 673–688. 12396583 10.1080/10635150290102366

[47] Rambaut A, Drummond AJ. Tracer v1.5. 2009. Available: http://beast.bio.ed.ac.uk/Tracer.

[48] Zwickl DJ. Genetic algorithm approaches for the phylogenetic analysis of large biological sequence datasets under the maximum likelihood criterion. Ph.D. Thesis, The University of Texas at Austin. 2006. Available: http://repositories.lib.utexas.edu/handle/2152/2666.

[49] Kumar S, Skjæveland Å, Orr RJS, Enger P, Ruden T, Mevik BH, et al (2009) AIR: A batch-oriented web program package for construction of supermatrices ready for phylogenomic analyses. BMC Bioinformatics 2009;10: 357 10.1186/1471-2105-10-357 19863793 PMC2777179

[50] Miller MA, Pfeiffer W, Schwartz T. Creating the CIPRES Science Gateway for inference of large phylogenetic trees Proceedings of the Gateway Computing Environments Workshop (GCE). 14 11 2010 pp. 1–8.

[51] Sukumaran J, Holder MT. DendroPy: A Python library for phylogenetic computing. Bioinformatics 2010;26: 1569–1571. 10.1093/bioinformatics/btq228 20421198

[52] Stöver BC, Müller KF. TreeGraph 2: Combining and visualizing evidence from different phylogenetic analyses. BMC Bioinformatics 2010;11: 7 10.1186/1471-2105-11-7 20051126 PMC2806359

[53] Pagel M, Meade A, Barker D. Bayesian estimation of ancestral character states on phylogenies. Syst Biol. 2004;53: 673–684. 15545248 10.1080/10635150490522232

[54] Paradis E, Claude J, Strimmer K. APE: analyses of phylogenetics and evolution in R language. Bioinformatics 2004;20: 289–290. 14734327 10.1093/bioinformatics/btg412

[55] R Development Core Team. R: A language and environment for statistical computing. R Foundation for Statistical Computing 2011 Available: http://www.R-project.org/. 10.1016/j.neuroimage.2011.01.013

[56] Bandelt HJ, Forster P, Röhl A. Median-joining networks for inferring intraspecific phylogenies. Mol Biol Evol. 1999;16: 37–48. 10331250 10.1093/oxfordjournals.molbev.a026036

[57] Dirks RM, Pierce NA. A partition function algorithm for nucleic acid secondary structure including pseudoknots. J Comput Chem. 2003;24: 1664–1677. 12926009 10.1002/jcc.10296

[58] Dirks RM, Pierce NA. An algorithm for computing nucleic acid base-pairing probabilities including pseudoknots. J Comput Chem. 2004;25: 1295–1304. 15139042 10.1002/jcc.20057

[59] Dirks RM, Bois JS, SchaeVer JM, Winfree E, Pierce NA. Thermodynamic analysis of interacting nucleic acid strands. SIAM Rev. 2007;49: 65–88.

[60] Zadeh N, Steenberg CD, Bois JS, Wolfe BR, Pierce MB, Khan AR, et al NUPACK: analysis and design of nucleic acid systems. J Comput Chem. 2011;32: 170–173. 10.1002/jcc.21596 20645303

[61] Huson DH, Bryant D. Application of phylogenetic network in evolutionary studies. Mol Biol Evol. 2006;23: 254–267. 16221896 10.1093/molbev/msj030

[62] Bruen TC, Philippe H, Bryant D. A simple and robust statistical test for detecting the presence of recombination. Genetics 2006;172: 2665–2681. 16489234 10.1534/genetics.105.048975 PMC1456386

[63] Kosakowsky Pond SL, Posada D, Gravenor MB, Woelk CH, Frost SDW. GARD: a genetic algorithm for recombination detection. Bioinformatics 2006;22: 3096–3098. 17110367 10.1093/bioinformatics/btl474

[64] Delport W, Poon AF, Frost SDW, Kosakovsky Pond SL. Datamonkey 2010: a suite of phylogenetic analysis tools for evolutionary biology. Bioinformatics 2010;26: 2455–2457. 10.1093/bioinformatics/btq429 20671151 PMC2944195

[65] Myllys M, Stenroos S, Thell A, Kuusinen M. High cyanobiont selectivity of epiphytic lichens in old growth boreal forest of Finland. New Phytol. 2007;173: 621–629. 17244057 10.1111/j.1469-8137.2006.01944.x

[66] Magain N, Serusiaux E. Do photobiont switch and cephalodia emancipation act as evolutionary drivers in the lichen symbiosis? A case study in the Pannariaceae (Peltigerales). PLOS ONE 2014;9: e89876 10.1371/journal.pone.0089876 24587091 PMC3933699

[67] Kimura M. The neutral theory of molecular evolution: A review of recent evidence. Jpn J Genet. 1991;66: 367–386. 1954033 10.1266/jjg.66.367

[68] Rudi K, Skulberg OM, Jakobsen KS. Evolution of cyanobacteria by exchange of genetic material among phyletically related strains. J Bacteriol. 1998;180: 3453–3461. 9642201 10.1128/jb.180.13.3453-3461.1998 PMC107303

[69] Tanabe Y, Kaya K, Watanabe MM. Evidence for recombination in the microcystin synthetase (*mcy*) genes of toxic cyanobacteria *Microcystis* spp. J Mol Evol. 2004;58: 633–641. 15461420 10.1007/s00239-004-2583-1

[70] Shishido TK, Kaasalainen U, Fewer DP, Rouhiainen L, Jokela J, Wahlsten M, et al Convergent evolution of [*D*-Leucine^1^] microcystin-LR in taxonomically disparate cyanobacteria. BMC Evol Biol. 2013;13: 86 10.1186/1471-2148-13-86 23601305 PMC3640908

