# Supplementary material for: Evolution of the tRNALeu (UAA) Intron and Congruence of Genetic Markers in Lichen-Symbiotic Nostoc
Source: PLoS One. 2015 Jun 22;10(6):e0131223. doi: 10.1371/journal.pone.0131223 (PMC4476775; doi:10.1371/journal.pone.0131223)
Supplement: S1 Table — List of specimens used in the study including collection information and NCBI GenBank accession numbers for the sequences. The sequences generated for this study are bolded. The collector and voucher information is presented only for newly generated sequences. (DOCX) [file pone.0131223.s004.docx]

**Table S1. Specimen information.** List of specimens used in the study including collection information and NCBI GenBank accession numbers for the sequences. The sequences generated for this study are bolded. The voucher information is presented for newly generated sequences.

**Accession Number**

**ID Species Host 16S rDNA *trnL* intron Collection location Voucher Herbarium**

"*Rhizonema*" sp. *Coccocarpia* sp. a **KF359679 KF359720** Kenya, Taita Hills JR111447 EA, Kenya

"*Rhizonema*" sp. *Coccocarpia* sp. b **KF359680 KF359721** Kenya, Taita Hills JR100073 EA, Kenya

"*Rhizonema*" sp. *Coccocarpia* sp. c **KF359678 KF359722** Kenya, Taita Hills JR10K254 EA, Kenya

1 *Nostoc* sp. *Sticta* cf. *caulescens*  **KF359681 KF359723** Argentina, Bariloche AR0311616 UPS, Sweden

2 *Nostoc* sp. *Peltigera britannica* **KF359682 KF359724** USA, Oregon^1^ UK08_152 *

3 *Nostoc* sp. *Fuscopannaria pacifica*  **KF359683 KF359725** USA, California^2^ UK08_179 *

4 *Nostoc* sp. *Protopannaria pezizoides*  **KF359684** HM448726 Finland, Kuhmo UK08_120 H, Finland

5 *Nostoc* sp. *Nephroma arcticum* **KF359685 KF359726** Finland, Saarijärvi JR08_B H, Finland

6 *Nostoc* sp. *Nephroma arcticum* **KF359686 KF359727** Finland, Saarijärvi UK10_564 H, Finland

7 *Nostoc* sp. *Nephroma arcticum* JQ007763 JN857088 Finland, Jehkats

8 *Nostoc* sp. *Nephroma expallidum*  AF506248 AJ571718 Finland, Lapland

9 *Nostoc* sp. *Protopannaria pezizoides* **KF359687 KF359728** Finland, Saarijärvi JR08_A H, Finland

10 *Nostoc* sp. *Nephroma arcticum* **KF359688 KF359729** Finland, Laukaa JR08_H18 H, Finland

11 *Nostoc* sp. *Peltigera malacea* **KF359689 KF359730** Finland, Autti UK06_019 H, Finland

12 *Nostoc* sp. *Peltigera malacea*  **KF359690 KF359731** Finland, Saarijärvi JR08_S H, Finland

13 *Nostoc* sp. *Peltigera aphthosa*  **KF359691 KF359732** Finland, Saarijärvi JR08_Z H, Finland

14 *Nostoc* sp. *Peltigera aphthosa* **KF359692 KF359733** Finland, Saarijärvi JR08_Y H, Finland

15 *Nostoc* sp. *Peltigera aphthosa* **KF359693 KF359734** Finland, Laukaa JR08_H7 H, Finland

16 *Nostoc* sp. *Peltigera aphthosa* **KF359694 KF359735** Finland, Kangasniemi UK08_078 H, Finland

17 *Nostoc* sp.  *Peltigera collina* **KF359695 KF359736** USA, Oregon^1^ UK08_144 *

18 *Nostoc* sp. *Leptogium palmatum*  JN847344 JN847370 USA, Oregon

19 *Nostoc* sp. *Leptogium palmatum* **KF359696 KF359737** USA, Oregon^3^ UK08_176 *

20 *Nostoc* sp. *Peltigera canina*  **KF359697** HM448741 Finland, Kuhmo UK08_121 H, Finland

21 *Nostoc* sp. *Peltigera membranacea* **KF359698 KF359738** USA, Oregon^1^ UK08_156 *

22 *Nostoc* sp. *Peltigera leucophlebia* **KF359699 KF359739** Finland, Saarijärvi JR08_H1 H, Finland

23 *Nostoc* sp. *Peltigera canina* **KF359700**  HM448736 Finland, Kuhmo UK08_106 H, Finland

24 *Nostoc* sp. *Peltigera canina* **KF359701**  HM448732 Finland, Kuhmo UK08_123 H, Finland

25 *Nostoc* sp. *Peltigera neopolydactyla* agg. **KF359702 KF359740** USA, Oregon^1^ UK08_150 *

26 *Nostoc* sp. *Peltigera* sp. JQ007800 **KF359741** Argentina, Bariloche AR1610211 UPS, Sweden

27 *Nostoc* sp. *Peltigera membranacea* **KF359703 KF359742** USA, California^4^ UK08_194 *

28 *Nostoc* sp. *Peltigera degenii* JQ007802 HM448725 Finland, Kuhmo

29 *Nostoc* sp. *Peltigera* sp. **KF359704 KF359743** Japan, Hokkaido AF 10/Jp1 UPS, Sweden

30 *Nostoc* sp. *Polychidium muscicola* **KF359705 KF359744** USA, Oregon^3^ UK08_175 *

31 *Nostoc* sp. *Leptogium pseudofurfuraceum* JN847347 JN847373 USA, California

32 *Nostoc* sp. *Leptogium gelatinosum* JN847337 JN847365 USA, Oregon

33 *Nostoc* sp. *Peltigera leucophlebia* **KF359706 KF359745** Finland, Saarijärvi JR08_Ö H, Finland

34 *Nostoc* sp. *Peltigera extenuata*  **KF359707 KF359746** Argentina, Bariloche AR1610111 UPS, Sweden

35 *Nostoc* sp. *Peltigera* sp. **KF359708 KF359747** Sweden, Ammarnäs UK09_437 H, Finland

36 *Nostoc* sp. *Peltigera neorufescens* JQ007762 **KF359748** Finland, Jehkats JR08J2C-J50 H, Finland

37 *Nostoc* sp. *Peltigera leucophlebia* FJ815291 **KF359749** Finland, Autti UK06_018aI H, Finland

38 *Nostoc* sp. *Peltigera dolichorhiza* JQ007769 **KF359750** Argentina, Bariloche AR0311711 UPS, Sweden

39 *Nostoc* sp. *Peltigera praetextata* JQ007767 **KF359751**  Scotland, Kindrogan UK09_409 H, Finland

40 *Nostoc* sp. *Peltigera rufescens*  **KF359709 KF359752** Finland, Helsinki JR080002 H, Finland

41 *Nostoc* sp. *Peltigera extenuata* **KF359710 KF359753** Finland, Helsinki UK06_004 H, Finland

42 *Nostoc* sp. *Leptogium furfuraceum* JQ007761 **KF359754** USA, California^5^ UK08_204 *

43 *Nostoc* sp. *Collema nigrescens* **KF359711 KF359755** USA, California^5^ UK08_197 *

44 *Nostoc* sp. *Collema* sp. **KF359712 KF359756** China, Hunan JR000734 H, Finland

45 *Nostoc* sp. *Peltigera collina* **KF359713 KF359757** USA, Oregon^1^ UK08_148 *

46 *Nostoc* sp. *Peltigera collina* **KF359714 KF359758** Scotland, Kindrogan UK09_407 H, Finland

47 *Nostoc* sp. *Peltigera evansiana* **KF359715 KF359759** USA, Oregon^1^ UK08_159 *

48 *Nostoc* sp. *Peltigera frigida* **KF359716 KF359760** Argentina, Bariloche AR1810211 UPS, Sweden

49 *Nostoc* sp. *Peltigera neopolydactyla* **KF359717 KF359761** Finland, Saarijärvi JR08_L H, Finland

50 *Nostoc* sp. *Peltigera scabrosa* **KF359718 KF359762** Finland, Saarijärvi JR08_I H, Finland

51 *Nostoc* sp. *Peltigera* cf. *degenii*  JQ007760 **KF359763** China, Hunan JR990942 H, Finland

52 *Nostoc* sp. *Leptogium furfuraceum* JN847353 JN847378 USA, California

53 *Nostoc* sp. *Collema nigrescens*  JN847352 JN847377 USA, California

54 *Nostoc* sp. *Collema nigrescens*  JN847348 JN847374 USA, California

55 *Nostoc* sp. *Collema* sp. **KF359719 KF359764** Argentina, Bariloche AR1910319 UPS, Sweden

56 *Nostoc* sp. *Leptogium* sp. JQ007740 **KF359765** Argentina, Bariloche AR1910521 UPS, Sweden

57 *Nostoc* sp. *Nephroma resupinatum* JN847335 JN847363 Finland, Saarijärvi

58 *Nostoc* sp. *Nephroma bellum* JN847334 JN847362 Finland, Saarijärvi

59 *Nostoc* sp. *Nephroma parile*  JN847360 JN857148 Norway, Vestfold

60 *Nostoc* sp. *Nephroma resupinatum* JN847336 JN847364 Finland, Laukaa

61 *Nostoc* sp. *Lobaria oregana* JN847338 JN847366 USA, Oregon

62 *Nostoc* sp. *Fuscopannaria leucostictoides* JN847350 JN847375 USA, California

63 *Nostoc* sp. *Lobaria virens* JN847358 JN847383 Norway, Vestfold

64 *Nostoc* sp. *Parmeliella triptophylla* JN847361 JN847384 Norway, Vestfold

65 *Nostoc* sp. *Sticta limbata* JN847351 JN857196 USA, California

66 *Nostoc* sp. *Lobaria retigera* AF506259 AF509408 China, Hunan

67 *Nostoc* sp. *Lobaria pulmonaria*  JN847357 JN847382 Norway, Vestfold

68 *Nostoc* sp. *Lobaria pulmonaria* JN847339 JN847367 USA, Oregon

69 *Nostoc* sp. *Nephroma washingtoniense* JN847340 JN857194 USA, Oregon

70 *Nostoc* sp. *Lobaria pulmonaria* JN847345 JN847371 USA, Oregon

71 *Nostoc* sp. *Nephroma washingtoniense* JN847341 JN857195 USA, Oregon

72 *Nostoc* sp. *Nephroma resupinatum* AF506263 AF509397 Finland, Saarijärvi

73 *Nostoc* sp. *Nephroma laevigatum* JN847359 JN857139 Norway, Vestfold

74 *Nostoc* sp. *Nephroma helveticum* JN847349 JN857135 USA, California

75 *Nostoc* sp. *Pseudocyphellaria* sp. JN847346 JN847372 USA, Oregon

76 *Nostoc* sp. *Pseudocyphellaria* sp. JN847355 JN847380 USA, California

77 *Nostoc* sp. *Sticta weigelii* JN847343 JN847369 USA, Oregon

78 *Nostoc* sp. *Sticta fuliginosa* JN847356 JN847381 USA, California

^1^ Willamette National Forest, Opal Creek Wilderness, 44°51’N, 122°15’W (collection permit issued by USDA Forest Service, Special Forest Products Coordinator John Poet).

^2^ East Weaver Creek Campground, 40°46’N, 122°55’W (collection permit issued by USDA Forest Service, Forest Officer Julie K. Nelson).

^3^ Upper Table Rock, 42°28’N, 122°53’W (collection permit issued by U.S. Department of the Interior Bureau of Land Management, Medford District Manager Mary Smelcer).

^4^ Patricks Point State Park, 41°08’N, 124°09’W (collection permit issued by California Department of Parks and Recreation, Natural Heritage Manager Carol Pehl).

^5^ Pepperwood Preserve, 38°34’N, 122°41’W (collection permit issued by California Department of Parks and Recreation, Natural Heritage Manager Carol Pehl).

* Collected for this study.
