# Supplementary material for: Evolution of the tRNALeu (UAA) Intron and Congruence of Genetic Markers in Lichen-Symbiotic Nostoc
Source: PLoS One. 2015 Jun 22;10(6):e0131223. doi: 10.1371/journal.pone.0131223 (PMC4476775; doi:10.1371/journal.pone.0131223)
Supplement: S2 Table — The numbering follows the numbering of the original Supporting Information S1 Table in O'Brien et al. [31]. The 'Identical trnL' column refers to the identical trnL sequences in the 16S rRNA gene–trnL data set. (DOCX) [file pone.0131223.s005.docx]

**Table S2. Data set of *rbcLX*, *nifV1*, *rpoC2*, and *trnL* from O'Brien et al. (2013).** The numbering follows the numbering of the original Supporting Information Table S1 in O'Brien et al. (2013). The 'Identical *trnL*' column refers to the identical *trnL* sequences in the 16S rRNA gene – *trnL* data set.

**NCBI GenBank Accession numbers Identical**

***rbcLX nifV1 rpoC2 trnL*  P6b type *trnL***

1 *Peltigera aphthosa* KC437655 KC437933 KC438054 KC438169 Collema -

2 *Peltigera aphthosa* KC437659 KC437934 KC438055 KC438170 Collema 47

7 *Peltigera malacea* KC437686 KC438024 KC438138 KC438254 Collema -

8 *Peltigera malacea* KC437687 KC438027 KC438140 KC438257 Collema 11

11 *Peltigera canina* KC437694 KC437941 KC438061 KC438177 Collema -

13 *Peltigera canina* KC437696 KC437943 KC438063 KC438179 Collema 47

16 *Peltigera fuscopraetextata* KC437712 KC437948 KC438068 KC438184 Collema -

19 *Peltigera fuscopraetextata* KC437715 KC437950 KC438070 KC438186 Collema -

24 *Peltigera fuscopraetextata* KC437720 KC438023 KC438137 KC438253 Collema -

27 *Peltigera* sp. A KC437727 KC438012 KC438126 KC438242 Collema -

28 *Peltigera neocanina* KC437735 KC438016 KC438130 KC438246 Class II 34

29 *Peltigera neocanina* KC437736 KC437924 KC438045 KC438160 Class II 23 & 24

36 *Peltigera horizontalis* KC437745 KC438029 KC438142 KC438259 Class II 23 & 24

45 *Peltigera kristinssonii* KC437754 KC437970 KC438090 KC438206 Collema -

63 *Peltigera leucophlebia* C. I KC437775 KC438013 KC438127 KC438243 Class II -

64 *Peltigera leucophlebia* C. I KC437776 KC437983 KC438103 KC438219 Collema 47

79 *Peltigera leucophlebia* C. III KC437822 KC437999 KC438118 KC438234 Collema 47

89 *Nephroma parile* KC437842 KC438017 KC438131 KC438247 Nephroma 57 & 59

90 *Nephroma bellum* KC437843 KC438032 KC438145 KC438262 Nephroma 58

107 *Nephroma arcticum* KC437886 KC438037 KC438154 KC438272 Collema 10

108 *Peltigera leucophlebia* C. III KC437888 KC438014 KC438128 KC438244 Collema -
