# Supplementary material for: Evolution of the tRNALeu (UAA) Intron and Congruence of Genetic Markers in Lichen-Symbiotic Nostoc
Source: PLoS One. 2015 Jun 22;10(6):e0131223. doi: 10.1371/journal.pone.0131223 (PMC4476775; doi:10.1371/journal.pone.0131223)
Supplement: S3 Table — P is the p-value of the PHI-test and RC the number of rate classes used in the GARD analysis. KH refers to Kishino Hasegawa test and * in the end of the row indicates that the GARD analysis stopped before convergence. 16S refers to the 16S rRNA gene. (DOCX) [file pone.0131223.s006.docx]

**Table S3. Results of the SplitsTree and GARD recombination tests.** P is the p-value of the PHI-test and RC the number of rate classes used in the GARD analysis. KH refers to Kishino Hasegawa test and * in the end of the row indicates that the GARD analysis stopped before convergence.

**SplitsTree GARD**

**KH p-values**

**Data set N taxa P RC Breakpoints LHS RHS**

16S 81 yes 0.0 3 589, 1031 0.00040 0.00040 *

16S reduced^1^ 17 - - 3 172, 1262 0.00080 0.00080 *

373 0.02400 0.00080

583 0.00480 0.07760

16S reduced^2^ 15 - - 3 94 0.00080 0.00040

1262 0.00040 0.00080

16S reduced^2^ 15 - - 5 94 0.00080 0.00040

1262 0.00040 0.00040

16S (no outgroup) 78 yes 0.0 3 573, 948 0.00040 0.00040 *

16S reduced (no outgroup)^3^ 16 - - 3 96 0.00080 0.00160

16S reduced (no outgroup)^3^ 16 - - 5 96 0.00060 0.00240

trnL 81 nd 0.3483 3 nd

trnL 81 - - 5 nd

trnL (*Nephroma*-type) 22 nd 0.1091 3 nd

trnL (*Collema*-type) 22 nd 0.6976 3 nd

trnL (Class II) 34 nd 0.9126 3 nd

trnL (all Class I) 44 nd 0.5606 3 nd

rbcLX (with insert) 21 yes 3.706E-5 3 212 0.03740 0.03100

rbcLX (without insert) 21 yes 2.027E-4 3 nd

nifV 21 yes 5.505E-4 3 nd

rpoC 21 yes 0.0404 3 nd

^1^ Included taxa: *Coccocarpia* a, 1, 5, 10, 15, 20, 25, 30, 35, 40, 45, 50, 55, 60, 65, 70, and 75.

^2^ Included taxa: *Coccocarpia* a, 1, 5, 10, 15, 20, 25, 30, 35, 45, 50, 55, 60, 70, and 75.

^3^ Included taxa: 1, 5, 10, 15, 20, 25, 30, 35, 40, 45, 50, 55, 60, 65, 70, and 75.
