# Supplementary material for: Evolution of the tRNALeu (UAA) Intron and Congruence of Genetic Markers in Lichen-Symbiotic Nostoc
Source: PLoS One. 2015 Jun 22;10(6):e0131223. doi: 10.1371/journal.pone.0131223 (PMC4476775; doi:10.1371/journal.pone.0131223)
Supplement: S4 Table — ID tells the taxa in which the P6b region is present. The secondary structure reconstruction and calculations were made with a sequence where two nucleotides outside the region were included in both ends, and in the ones marked with the * six nucleotides from the beginning and seven from the end outside the P6b region were included to ensure the correct folding [17]. (DOCX) [file pone.0131223.s007.docx]

**Table S4. *trnL* P6b region secondary structure reconstruction.** ID tells the taxa in which the P6b region is present. The secondary structure reconstruction and calculations were made with a sequence where two nucleotides outside the region were included in both ends, and in the ones marked with the * six nucleotides from the beginning and seven from the end outside the P6b region were included to ensure the correct folding [17].

**Free energy (20 °C)**

**ID Type Length (nts) E (kcal/mol) E /nt**

1 Class II 83 -35.93 -0.41

2 Class II 83 -39.44 -0.45

3 Class II 80 -30.12 -0.36

4 Class II 82 -31.81 -0.37

6 Class II 79 -31.97 -0.39

7 Class II 80 -28.42 -0.34

9 Class II 79 -31.97 -0.39

17 Class II 65 -25.47 -0.37

20 Class II 81 -34.88 -0.41

21 Class II 81 -38.76 -0.46

22 Class II 81 -33.65 -0.40

23, 24 Class II 81 -34.56 -0.41

25 Class II 74 -31.02 -0.40

26 Class II 80 -27.16 -0.32

27, 28 Class II 52 -22.04 -0.39

29 Class II 82 -36.17 -0.42

33 Class II 81 -34.44 -0.41

34, 36 Class II 81 -34.36 -0.40

35 Class II 53 -24.13 -0.42

37 Class II 81 -34.56 -0.41

39 Class II 81 -34.36 -0.40

40 Class II 81 -31.59 -0.37

41 Class II 74 -31.47 -0.40

42 Class II 82 -32.49 -0.38

43 Class II 82 -35.22 -0.41

44 Class II 82 -33.56 -0.39

45 Class II 82 -36.12 -0.42

46 Class II 82 -35.22 -0.41

49 Class II 67 -32.63 -0.46

50 Class II 74 -31.02 -0.40

51 Class II 80 -32.19 -0.38

5, 13–15 Collema 50 -18.70 -0.35

8 Collema 78 -29.66 -0.36

10 Collema 78 -29.61 -0.36

11 Collema 64 -23.30 -0.30 *

12 Collema 88 -32.49 -0.32 *

16 Collema 78 -29.73 -0.36

18, 19 Collema 64 -22.91 -0.34

30 Collema 126 -47.06 -0.36

31, 32 Collema 78 -29.77 -0.36

38 Collema 78 -34.94 -0.43

47, 48 Collema 64 -21.08 -0.27 *

52, 54 Collema 77 -32.03 -0.36 *

53 Collema 122 -53.25 -0.39 *

55 Collema 122 -49.81 -0.37 *

56 Collema 78 -34.02 -0.37 *
